# Functional multi-organelle units control inflammatory lipid metabolism of macrophages

**DOI:** 10.1038/s41556-024-01457-0

**Published:** 2024-07-05

**Authors:** Julia A. Zimmermann, Kerstin Lucht, Manuel Stecher, Chahat Badhan, Katharina M. Glaser, Maximilian W. Epple, Lena R. Koch, Ward Deboutte, Thomas Manke, Klaus Ebnet, Frauke Brinkmann, Olesja Fehler, Thomas Vogl, Ev-Marie Schuster, Anna Bremser, Joerg M. Buescher, Angelika S. Rambold

**Affiliations:** 1 Max Planck Institute of Immunobiology and Epigenetics, Freiburg, Germany; 2 Center of Chronic Immunodeficiency, Medical Center University of Freiburg, Freiburg, Germany; 3 International Max Planck Research School for Immunobiology, Epigenetics and Metabolism, Freiburg, Germany; 4 Faculty of Biology, University of Freiburg, Freiburg, Germany; 5 Bioinformatics Core Facility, Max Planck Institute of Immunobiology and Epigenetics, Freiburg, Germany; 6 Institute-Associated Research Group: Cell Adhesion and Cell Polarity, Institute of Medical Biochemistry, ZMBE, University of Munster, Munster, Germany; 7 Institute of Immunology, University of Munster, Munster, Germany; 8 Metabolomics Core Facility, Max Planck Institute of Immunobiology and Epigenetics, Freiburg, Germany

**Keywords:** Energy metabolism, Monocytes and macrophages, Membrane fission, Peroxisomes, Endoplasmic reticulum

## Abstract

Eukaryotic cells contain several membrane-separated organelles to compartmentalize distinct metabolic reactions. However, it has remained unclear how these organelle systems are coordinated when cells adapt metabolic pathways to support their development, survival or effector functions. Here we present OrgaPlexing, a multi-spectral organelle imaging approach for the comprehensive mapping of six key metabolic organelles and their interactions. We use this analysis on macrophages, immune cells that undergo rapid metabolic switches upon sensing bacterial and inflammatory stimuli. Our results identify lipid droplets (LDs) as primary inflammatory responder organelle, which forms three- and four-way interactions with other organelles. While clusters with endoplasmic reticulum (ER) and mitochondria (mitochondria–ER–LD unit) help supply fatty acids for LD growth, the additional recruitment of peroxisomes (mitochondria–ER–peroxisome–LD unit) supports fatty acid efflux from LDs. Interference with individual components of these units has direct functional consequences for inflammatory lipid mediator synthesis. Together, we show that macrophages form functional multi-organellar units to support metabolic adaptation and provide an experimental strategy to identify organelle-metabolic signalling hubs.

## Abstract (web summary)

Zimmermann et al. present OrgaPlexing, an imaging pipeline mapping metabolic organelles and their interactions. They find changes in mitochondria, ER, peroxisome and lipid droplet dynamics that impact macrophage inflammatory lipid mediator synthesis.

## Main

Organelle communication is critical to connect metabolic pathways, whose subprocesses are segregated in different organelles across the cell. Lipid metabolism, for example, involves (1) lipid transport and storage in lipid droplets (LDs), (2) β-oxidation in mitochondria and peroxisomes, (3) lipid breakdown and recycling in lysosomes, and (4) lipid synthesis in the endoplasmic reticulum (ER), peroxisomes and mitochondria. Organelle communication has primarily been studied between two organelles, which was the basis for the identification of tether proteins mediating direct inter-organellar contacts^1^. From such studies, it has become clear that most organelles can theoretically interact with most other organelles^1^. However, whether and how cells simultaneously coordinate the dynamics and functions of multiple organelle systems to fully integrate complex metabolic pathways is unclear. This is particularly relevant for cells that undergo metabolic switches to adapt their cellular state or functions. Macrophages can rapidly reprogramme metabolic pathways to sustain their cellular bioenergetics and instruct antimicrobial effector functions^2,3^. Upon activation with bacterial and inflammatory stimuli, they rapidly upregulate glycolysis, reduce mitochondrial respiration, rewire the tricarboxylic acid cycle fuelling and remodel their lipid metabolism^4,5^. Previous imaging studies showed that this metabolic activation is accompanied by changes in individual organelle systems^6–8^. However, the overall adaptations of the cellular organellome have not been addressed. The simultaneous visualization of six organelles has only been mastered once in non-immune cell lines, yet without providing functional insight into the observed organelle clusters^9^. For primary immune cells, a system-wide organelle imaging approach has not yet been attempted due to its technical limitations. Thus, we are still missing clear concepts on the combined response of all organelle systems (organelle network response) within cells and especially its importance for immune cells, whose functions critically depend on metabolic adaptation.

## Results

### OrgaPlexing defines organellar adaptations upon macrophage activation

**Fig. 1:**
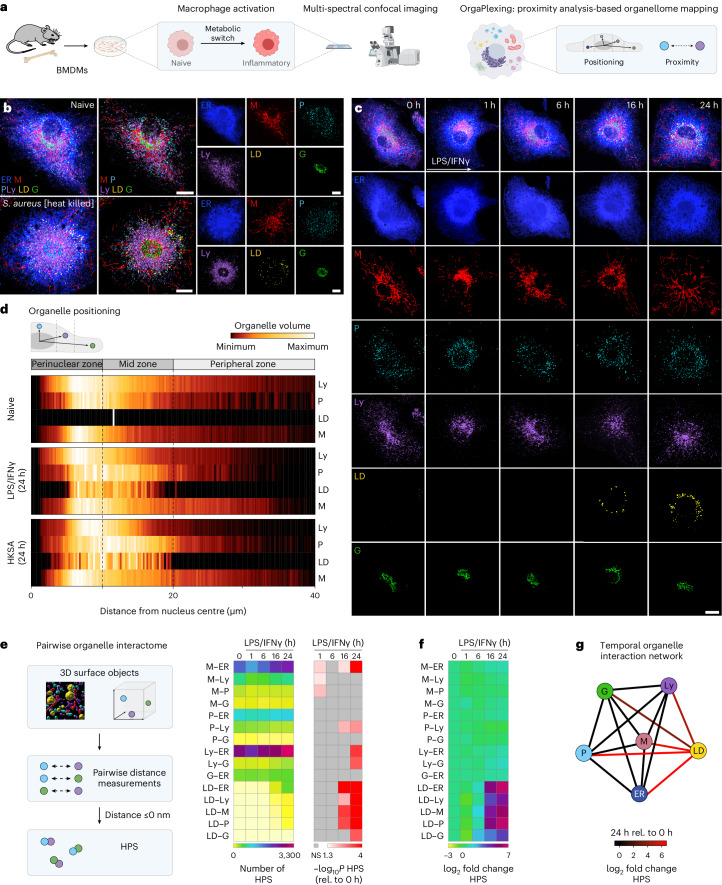
Inflammatory stimuli rewire the macrophage organellome. **a**, Scheme representing the OrgaPlexing work flow. **b**,**c**, Immunofluorescence images showing the BMDM organellome upon heat-killed *S. aureus* (24 h) (**b**) or LPS/IFNγ (0–24 h) treatment (**c**). The organelles were visualized using ERmoxVenus (ER, blue), HSP60 (mitochondria (M), red), catalase (peroxisomes (P), cyan), LAMP1 (lysosomes (Ly), purple), Bodipy^493/502^ (LDs, yellow) and GM130 (Golgi body (G), green). The images are maximum intensity projections and are representative of *n* = 3 (**b**,**c**, 16 h) and *n* = 4 (**c**, 0–6 and 24 h) independent experiments. Scale bars, 10 µm. **d**, Heat maps representing the organelle distribution in BMDMs upon inflammatory activation of *N* = 15 cells examined over *n* = 3 independent experiments. The dotted lines indicate borders of perinuclear, mid and peripheral zones. **e**, Work flow and heat maps showing the number of pairwise organelle HPS upon LPS/IFNγ treatment. Data represent the mean of *N* = 59 (0 h), *N* = 48 (1 h), *N* = 56 (6 h), *N* = 46 (16 h) and *N* = 59 (24 h) cells obtained from *n* = 3 (16 h) and *n* = 4 (0–6 and 24 h) independent experiments. *P* values were obtained using one-way analysis of variance (ANOVA) with Tukey’s post hoc test and are depicted as −log_10_. **f**, log_2_-transformed fold change of pairwise organelle proximity sites as described in **e**. **g**, Network graph representing the log_2_-transformed fold change of pairwise organelle proximity sites of LPS/IFNγ-treated (24 h) BMDMs relative (rel.) to naive cells. The numerical *P* values are indicated in Supplementary Table 4. *P ≤ 0.05, **P ≤ 0.01, ***P ≤ 0.001, ****P ≤ 0.0001. NS, not significant (*P* > 0.05). Source numerical data are available in Source data. Source data

**Extended Data Fig. 1:**
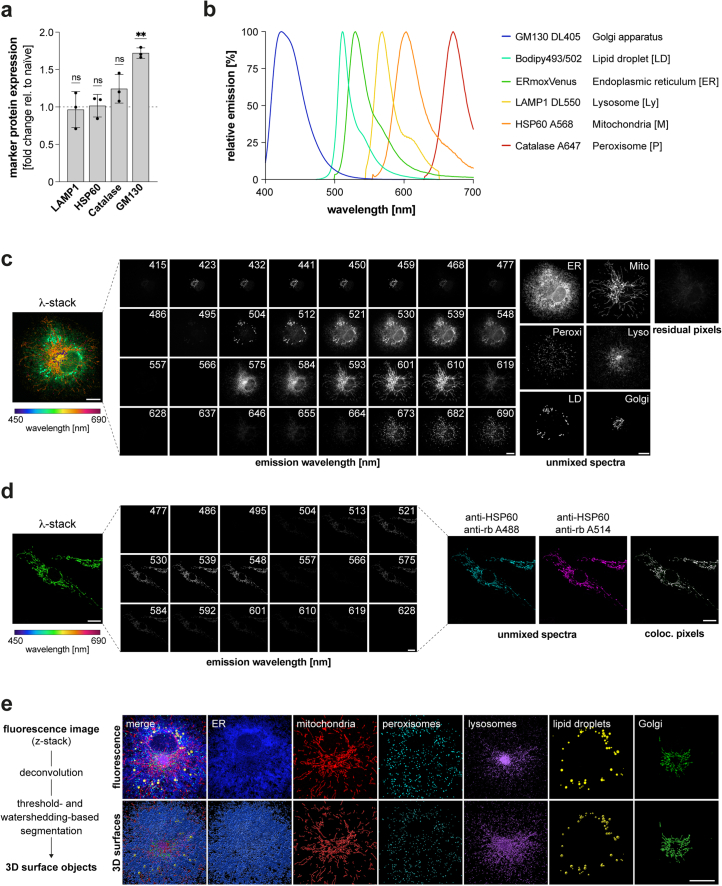
Spectral imaging strategy, image processing and segmentation. **a** Expression of marker proteins used for spectral imaging in LPS/IFN-γ-treated (24 h) BMDMs relative to the control obtained by flow cytometry analysis. Bars show the mean ± SD of n = 3 biologically independent experiments. *P* values were obtained by two-tailed, one-sample *t* test (** *P* ≤ 0.01, not significant (ns) *P* >0.05). Numerical *P* values are indicated in Supplementary Information Table 4. **b** Emission spectra of fluorophores used for OrgaPlexing. **c** Immunofluorescence images of a 6-colour labelled BMDM displaying the emission spectra of individual organelles before (left) and after (right) spectral unmixing. All images are maximum intensity projections and representative of n = 4 independent experiments. Scale bars: 10 µm. **d** Verification of the unmixing strategy, shown by immunofluorescence of a BMDM displaying mitochondria (HSP60) labelled with A488 and A514 before (left, middle panel) and after (right, unmixed spectra) spectral unmixing as well as colocalized pixels between unmixed images (right, coloc. pixels). All images represent a single z-plane and representative of n = 3 experimental repeats. Scale bars: 10 µm. **e** Scheme depicting image processing and segmentation pipeline and images showing 3D surface objects generated for each organelle. Fluorescence images show maximum intensity projections and are representative of n = 4 independent experiments. Scale bar: 10 µm. Source numerical data are available in source data. Source data

To examine the spatiotemporal coordination of organelle dynamics in primary macrophages, we established OrgaPlexing, a multi-spectral organelle imaging approach that allows the simultaneous visualization and behavioural analysis of up to six organelles (Fig. 1a). We used this methodology to study the organellome response of bone marrow-derived mouse macrophages (BMDMs) responding to heat-killed *Staphylococcus aureus* or lipopolysaccharide (LPS)/interferon-γ (IFNγ) (Fig. 1a–c). BMDMs were transduced with an ER-targeted fluorescent protein (KDEL-mOX-Venus) (Fig. 1b,c), before undergoing additional fluorescent dye-labelling of LDs and antibody-based immunostainings for peroxisomes (catalase), mitochondria (HSP60), Golgi apparatus (GM130) and lysosomes (LAMP1) (Fig. 1b,c and Extended Data Fig. 1a–c). This stringently validated antibody panel enabled the highly specific organelle labelling in activated macrophages (Fig. 1a,b and Extended Data Fig. 1c,d). Images were acquired in lambda mode using a laser-scanning fluorescence confocal microscope with a spectral detector, and the overlapping fluorescent spectra were unmixed resulting in six fluorophore compartments (Extended Data Fig. 1c). By combining multi-spectral imaging with advanced three-dimensional (3D)-image analysis (Extended Data Fig. 1e), we generated a workflow for the time-resolved analysis of organelle mass, positioning and organellar interactions in macrophages.

**Extended Data Fig. 2:**
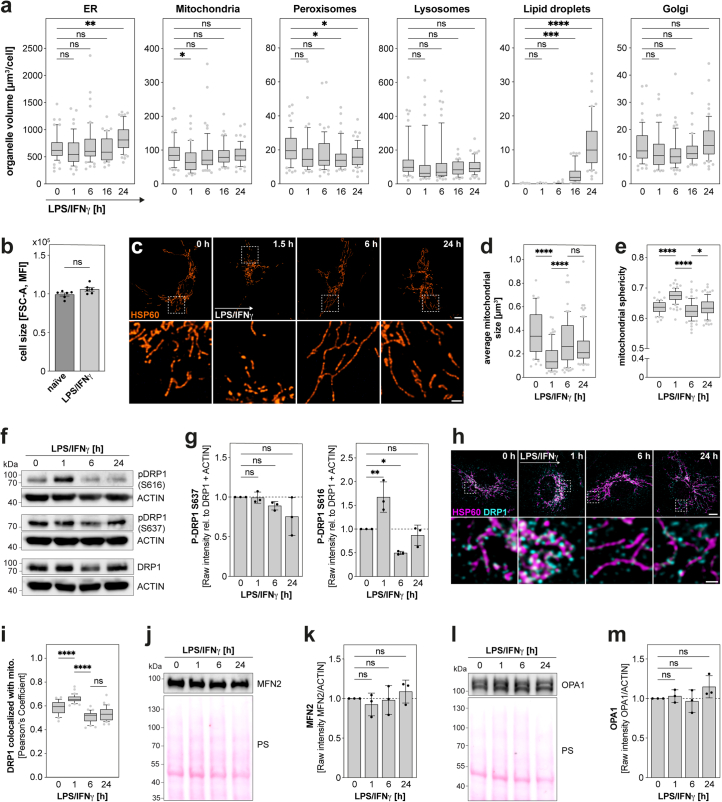
Organelle mass and mitochondrial morphology are selectively altered in inflammatory macrophages. **a** Quantification of organellar volume upon LPS/IFN-γ treatment. Data represent N = 59 (0 h), N = 48 (1 h), N = 56 (6 h), N = 46 (16 h), N = 59 (24 h) cells from n = 3 (16 h, LD 0–6 h) and n = 4 (non-LD 0–6, 24 h) independent experiments. *P* values were calculated using one-way ANOVA with Dunnett’s post-hoc test. **b** Cell size of naïve and 24h LPS/IFN-γ-treated BMDMs representing n = 6 independent experiments. *P* value was obtained by two-tailed, unpaired *t*-test. **c-e** Images (**c**) and quantification (**d**, average mitochondrial volume/cell; **e**, mitochondrial sphericity) showing dynamics in mitochondrial morphology of LPS/IFN-γ-activated BMDMs. Images are single z-planes and representative of n = 3 independent experiments. Scale bars: 5 µm, 1 µm (magnification) (**c**). Data represent (**d**) N = 51 (0 h), N = 76 (1 h), N = 86 (6 h) and N = 80 (24 h) and (**e**) N = 55 (0 h), N = 78 (1 h), N = 71 (6 h), N = 79 (24 h) cells from n = 3 independent experiments. *P* values were calculated using one-way ANOVA with Tukey’s post-hoc test. **f-g** Western blot analysis (**f**) and quantification (**g**) showing protein levels and activation state of DRP1 upon LPS/IFN-γ treatment. Data represent n = 3 biological replicates. *P* values were calculated using one-way ANOVA with Sidak’s post-hoc test (**g**). **h,i** Images (**h**) and quantification (**i**) showing the localization of DRP1 to mitochondria (HSP60) in LPS/IFN-γ-activated BMDMs. Scale bars: 5 µm, 1 µm (magnification) (**h**). Data represent N = 26 (0 h), N = 32 (1 h), N = 30 (6 h), N = 30 (24 h) cells from n = 3 biological replicates. *P* values were calculated using one-way ANOVA with Tukey’s post-hoc test (**i**). **j-m** WB analysis (**j,l**) and quantification (**k,m**) showing protein levels of MFN2 (**j-k**) and OPA1 (**l-m**) upon LPS/IFN-γ treatment. Data represent n = 3 biological replicates. *P* values were calculated using one-way ANOVA with Sidak’s post-hoc test (**k,m**). Boxes represent 25^th^ to 75^th^ percentiles, whiskers 10^th^ and 90^th^ percentiles. Dots are outliers, median is shown as line (**a,d,e,i**). The bars show the mean ± SEM (**b**) or the mean ± SD (**g,k,m**). Numerical *P* values are indicated in Supplementary Information Table 4 (*P ≤ 0.05, **P ≤ 0.01, ***P ≤ 0.001, ****P ≤ 0.0001, not significant (ns) *P* > 0.05). Source numerical data and unprocessed blots are available in source data. Source data

OrgaPlexing confirmed previously described changes on the single organelle level, including the substantial accumulation of LDs in response to bacterial and inflammatory signals^6^ (Fig. 1b,c and Extended Data Fig. 2a). Additionally, our refined temporal analysis revealed mild changes in ER and peroxisomal volume that occurred at late stages of LPS/IFNγ stimulation (Extended Data Fig. 2a, 16–24 h), independent of cell size (Extended Data Fig. 2b). Mitochondria, reported to undergo fission early upon macrophage activation^7,8^ (Fig. 1c and Extended Data Fig. 2c–e), were found to re-tubulate by 16–24 h (Fig. 1c and Extended Data Fig. 2c–e). This late mitochondrial remodelling was accompanied by decreased levels of phosphorylated DRP1^S616^ (Extended Data Fig. 2f,g), the active form of the major mitochondrial fission factor DRP1^10^, and reduced DRP1-mitochondria co-localization (Extended Data Fig. 2h,i). In contrast to the regulation of DRP1, key mitochondrial fusion factors remained unchanged (Extended Data Fig. 2j–m).

**Extended Data Fig. 3:**
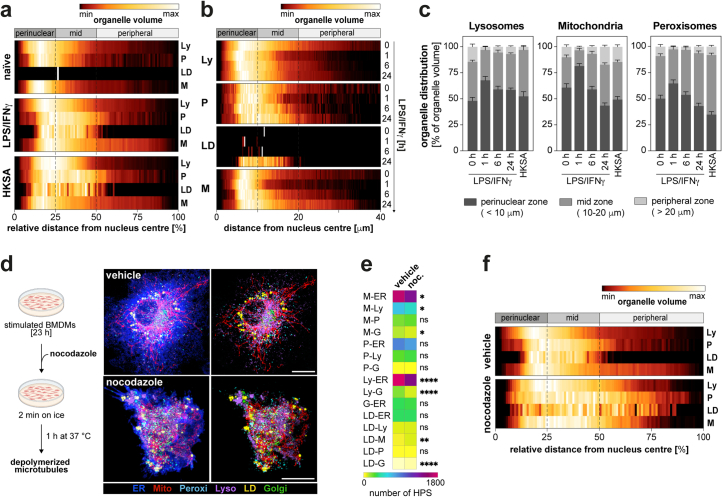
Cellular positioning of organelles is rewired upon inflammatory macrophage activation. **a** Heatmaps representing the organelle distribution relative to the maximal distance from nucleus centre in BMDMs upon inflammatory activation (24 h LPS/IFN-γ or heat-killed *S. aureus*). Data are representative for N = 15 cells examined over n = 3 independent experiments. Dotted lines indicate borders of perinuclear, mid and peripheral zones. **b** Heatmaps depicting time-resolved organelle positioning upon LPS/IFN-γ treatment. Data represent the mean of N = 15 cells examined over n = 3 independent experiments. Dotted lines indicate borders of perinuclear, mid and peripheral zones. **c** Quantification of organelle distribution in perinuclear, mid and peripheral zones in BMDMs activated with LPS/IFN-γ (1–24 h) or heat-killed *S. aureus* (24 h). Bars show mean ± SEM of N = 15 cells examined over n = 3 independent experiments. **d** Immunofluorescence images depicting the organellome of LPS/IFN-γ-activated BMDMs (24 h) upon nocodazole treatment. Organelles were visualized using ERmoxVenus (ER, blue), HSP60 (mitochondria, red), Catalase (peroxisomes, cyan), LAMP1 (lysosomes, purple), Bodipy^493/502^ (lipid droplets, yellow) and GM130 (Golgi, green). Images are maximum intensity projections and are representative of n = 2 biological repeats. Scale bars: 10 µm. **e-f** Heatmaps showing changes in pair-wise organelle proximity sites (**e**) and organelle distribution (**f**) upon nocodazole treatment. Data represent the mean of N = 30 (**e**) and N = 15 (**f**) cells over n = 2 biological experiments (**e-f**). *P* values were obtained by two-tailed, unpaired *t*-test (* *P* ≤ 0.05, ** *P* ≤ 0.01, *** *P* ≤ 0.001, not significant (ns) *P* > 0.05) (**e**). Numerical *P* values are available in Supplementary Information Table 4. Source numerical data are available in source data. Source data

Systematic inter-organelle mapping revealed the full extent of the rewired organellar architecture upon macrophage activation. When treated with LPS/IFNγ or heat-killed *S. aureus*, macrophages dynamically adapted their organelle positioning relative to the nucleus (Fig. 1b–d and Extended Data Fig. 3a–c). After 24 h, lysosomes preferentially inhabited the perinuclear zone of fully activated cells, whereas peroxisomes spread into the cellular midzone, overlapping with the territory of accumulating LDs (Fig. 1d and Extended Data Fig. 3a). Tubulated mitochondria spread from the perinuclear zone to the cell periphery and permeated the peroxisome–LD (P–LD)-rich midzone (Fig. 1d and Extended Data Fig. 3a). To also analyse how different organelle systems localize to each other, we next performed a pairwise proximity analysis of all potential 15 organelle pairs (Fig. 1e). We defined measured distances of ≤0 nm between the fluorescent signals of two organelles as organellar high-proximity sites (HPS). Owing to the resolution limit of confocal microscopy in combination with deconvolution, HPS cover inter-organelle distances of <125 nm in *xy* and <270 nm in *z*, thus including the range of most direct organelle contact sites, as well as organelle proximities without direct contact^11^. Total HPS numbers in naive macrophages (0 h) already revealed a range of proximity relations between organelle pairs (Fig. 1e). Notably, activation of macrophages with LPS/IFNγ for 24 h induced substantial changes in HPS numbers across 9 out of 15 organelle pairs (Fig. 1e). Interactions of the ER with mitochondria (M–ER) and lysosomes were increased, whereas interactions between peroxisomes and lysosomes were decreased upon macrophage activation. LDs were identified as strongest inflammatory responders, showing the most substantial increases of HPS numbers with several other organelles (Fig. 1f,g). As expected, the rewiring of organelle interactions required a functional microtubule network. Treatment with the microtubule-depolymerizing drug nocodazole led to broad reductions in organelle interactions and eliminated organelle zonation in inflammatory macrophages (Extended Data Fig. 3d–f), confirming the functional roles of microtubules for organelle positioning and interactions^12^. Thus, our mapping data revealed that macrophages actively and dynamically remodel their organelle architecture when responding to bacterial or inflammatory signals. Notably, OrgaPlexing highlighted LDs and the LD–organelle interactome as primary responders to inflammatory macrophage activation.

### In activated macrophages LDs form clusters with mitochondria, ER and peroxisomes

**Fig. 2:**
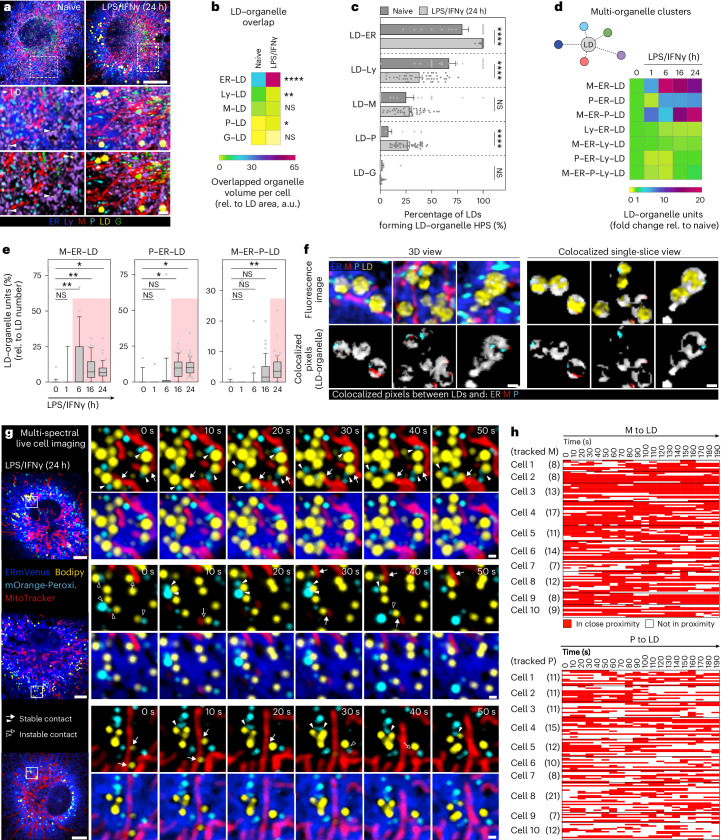
Macrophages rewire their LD–interactome in response to inflammatory stimuli by selectively assembling M–ER–P–LD units. **a**, Images of LDs and surrounding organelles in presence or absence of LPS/IFNγ (24 h). The arrowheads are the LDs in naive BMDMs. The organelles were visualized as described in (Fig. 1b,c). The images are maximum intensity projections and representative of *n* = 3 independent experiments. Scale bars, 5 µm (top) and 1 µm (bottom). **b**,**c**, Total overlapped volume between LDs and respective organelles relative to total LD surface area per cell (**b**) and pairwise LD–organelle HPS relative to LD number (**c**) in naive and LPS/IFNγ-treated (24 h) BMDMs. The data represent *N* = 19 (naive) and *N* = 51 (LPS/IFNγ) cells from *n* = 3 independent experiments. *P* values were obtained using Brown–Forsythe and Welch ANOVA with Dunnett’s post hoc (**b**) or one-way ANOVA with Sidak’s post hoc tests (**c**). Data are mean ± s.e.m. (**c**). a.u., arbitrary unit. **d**, LD-centric multi-organelle clusters upon LPS/IFNγ treatment (0–24 h). The data represent *N* = 17 (0 h, 1 h), *N* = 21 (6 h), *N* = 44 (16 h) and *N* = 51 (24 h) cells from *n* = 3 independent experiments. **e**, Upregulated LD multi-organelle clusters in inflammatory macrophages (as shown in **d**). The boxes represent the 25th to 75th percentiles and the whiskers denote the 10th and 90th percentiles. The dots are the outliers, and the median is shown as a line. The red colouring indicates significant changes. *P* values were generated using one-way ANOVA with Dunnett’s post hoc test. **f**, Co-localized pixels of LDs and respective organelles are depicted as a 3D overlay (left) or single *z*-planes (right). The data are representative of *n* = 3 independent experiments. Scale bars, 0.5 µm. **g**, Mitochondria–LD (arrows) and P–LD (arrowheads) interaction dynamics in LPS/IFNγ-treated BMDMs (24 h). The stable interactions are indicated with filled, instable interactions with empty arrows or arrow heads, respectively. The images represent single confocal *z*-planes from *n* = 3 biological repeats. Scale bars, 5 µm (left) and 0.5 µm (right). **h**, Mitochondria–LD (top) and P–LD (bottom) contacts. The rows represent individual organelles that are (red) or are not (white) in close proximity to LDs. The data representing *N* = 107 mitochondria and *N* = 118 peroxisomes were tracked across ten cells from *n* = 3 biological replicates. Numerical *P* values are available in Supplementary Table 4. *P ≤ 0.05, **P ≤ 0.01, ***P ≤ 0.001, ****P ≤ 0.0001. Source numerical data are available in Source data. Source data

**Extended Data Fig. 4:**
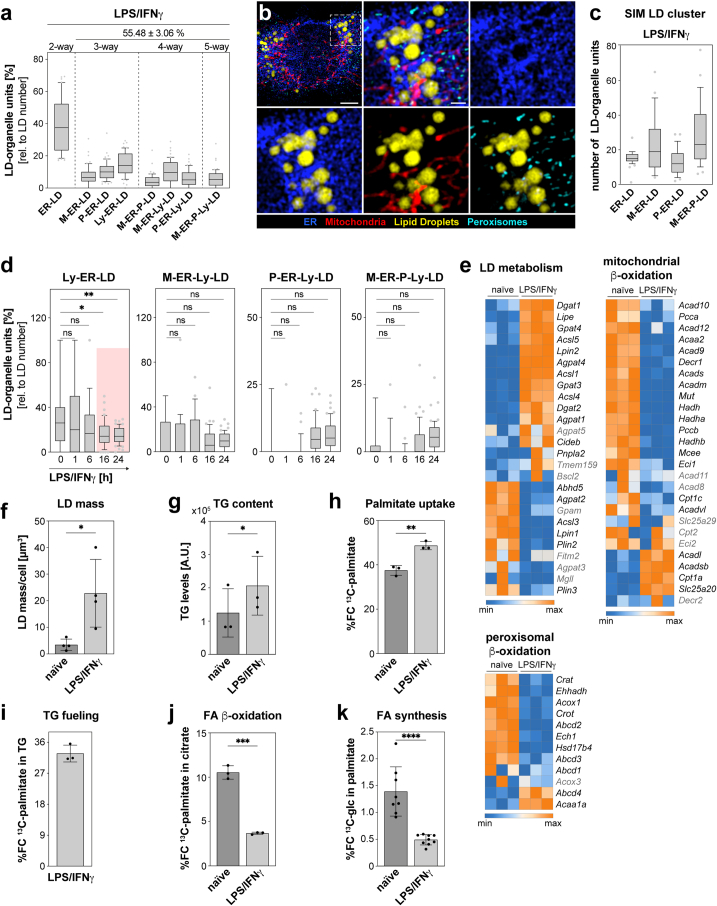
LD metabolism and the LD interactome is modified upon inflammatory macrophage activation. **a** LD-organelle clusters identified in inflammatory macrophages (24 h). Boxes represent 25^th^ to 75^th^ percentiles, whiskers 10^th^ and 90^th^ percentiles. Dots are outliers, the median is shown as line. The percentage of LDs associated in multi-organelle clusters is depicted as mean ± SEM (top). Data represent N = 51 cells from n = 3 independent experiments. **b** Structured illumination images showing LDs and their surrounding organelles in LPS/IFN-γ-treated (24 h) BMDMs. Organelles were visualized using Calnexin (ER, blue), HSP60 (mitochondria, red), Catalase (peroxisomes, cyan) and Bodipy^493/502^ (LDs, yellow). Images are single LD-containing z-slices. Scale bars: 3 µm (top), 1 µm (bottom). **c** Quantification of LD-organelle clusters shown in (**b**). Data represent of N = 30 cells from n = 3 biological replicates. Boxes represent 25^th^ to 75^th^ percentiles, whiskers 10^th^ and 90^th^ percentiles. Dots are outliers, the median is shown as line. **d** LD multi-organelle units that are downregulated or not significantly altered upon LPS/IFN-γ treatment (as shown in (Fig. 2e)). Boxes represent 25^th^ to 75^th^ percentiles, whiskers 10^th^ and 90^th^ percentiles. Dots are outliers, the median is shown as line. Red colouring indicates significant changes. *P* values were generated using one-way ANOVA with Dunnett’s post-hoc test. **e** RNA-Sequencing analysis of LD metabolism- and b-oxidation-associated genes in naïve and LPS/IFN-γ-treated (24 h) BMDMs. Data represent N = 3 technical replicates from n = 1 biological replicate. Black font: *P* ≤ 0.05, grey font: ns *P* > 0.05. **f-j** Quantification of LD mass (**f**), cellular triglyceride (TG) content (**g**), cellular ^13^C-palmitate uptake (**h**), ^13^C-palmitate content in TG (**i**) and ^13^C palmitate oxidation (**j**) in LPS/IFN-γ-activated (24 h) BMDMs relative to controls. Data represent n = 4 (**f**) and n = 3 (**g-j**) biological replicates. *P* values were calculated using two-tailed unpaired (**f, h, j**) and paired (**g**) *t* tests. **k** ^13^C-glucose tracing analysis showing the contribution of glycolysis to fatty acid (FA) synthesis in the presence or absence of LPS/IFN-γ (24h). Data represent n = 8 (naïve) and n = 9 (LPS/IFN-γ) independent biological replicates. *P* value was obtained using two-tailed, unpaired t test. All bars show mean ± SD (**f-k**). Numerical *P* values are available in Supplementary Information Table 4 (*P ≤ 0.05, **P ≤ 0.01, ***P ≤ 0.001, ****P ≤ 0.0001, not significant (ns) *P* > 0.05). Source numerical data are available in source data. Source data

Having identified LDs as strongest responding organelle system in fully activated macrophages, we next asked whether the increased LD-associated organelle interactions were solely attributed to the noticeable increase in LD mass and size (Figs. 1c,e–f, 2a and Extended Data Fig. 2a). Normalizing the total LD–organelle overlap to LD surface area confirmed that the inflammatory LD interactome is formed at least partially independent of the expanding LD mass (Fig. 2b). Furthermore, normalizing HPS numbers to LD numbers unveiled that the inflammatory interactome also undergoes dynamic adaptations for each individual LD (Fig. 2c). Given that organelles have the potential to simultaneously contact multiple other compartments^1^, we next addressed whether LPS/IFNγ-driven LD-associated organelle interactions involve more than two-way interactions, by expanding our proximity analysis to up to six-way interactions (Fig. 2d,e). In inflammatory macrophages, we found that more than 50% of all LDs were part of clusters with three or more other organelles (Extended Data Fig. 4a). Temporal multi-way LD-interactome analysis revealed that inflammatory macrophages induced LDs to build selective ‘mitochondria–ER–peroxisome–LD (M–ER–P–LD)’ clusters that contained the ER, the organelle-site of LD biogenesis^13^, together with mitochondria and/or peroxisomes (Fig. 2d). The formation of these clusters could be confirmed by structured illumination microscopy (Extended Data Fig. 4b,c). In contrast to M–ER–P–LD, organelle clusters containing lysosomes remained either unchanged or were down-regulated in inflammatory (24 h) versus naive (0 h) BMDMs (Fig. 2d and Extended Data Fig. 4d). Detailed analysis of different M–ER–P–LD clusters revealed a specific assembly time course: M–ER–LD formed first at 6 h, followed by P–ER–LD at 16 h and M–ER–P–LD at 24 h after LPS/IFNγ stimulation (Fig. 2e). Although still unclear, this temporal order may suggest differential contributions of the distinct organelle clusters to LD accumulation and function. Interestingly, individual organelles of M–ER–P–LD showed specific interaction characteristics. Mitochondria often contacted LDs close to ER–LD interactions (Fig. 2f), indicating potential mitochondria–ER contact sites. Live cell imaging at these sites identified sustained interactions for up to 180 s between mitochondria and LDs (Fig. 2g,h). Conversely, peroxisomes showed more heterogeneous interaction profiles (Fig. 2g) and overall shorter LD interaction times in comparison with mitochondria (Fig. 2g,h). Occasionally, P–LD contacts showed rapid on–off cycles, suggesting transitions between peroxisome-containing versus peroxisome-devoid LD clusters (Fig. 2g,h). Thus, our data demonstrate that inflammatory macrophage signalling induces coordinated organelle dynamics and clustering, which result in the formation of LD-containing units of three to four organelles as part of the activation response.

### Macrophages utilize clustered organelles for inflammatory lipid trafficking

LDs are the central organelle system of lipid metabolism and fulfil important innate immune functions in macrophages^6,14,15^. In line with previous reports, we show that inflammatory macrophages markedly alter their lipid metabolism at the transcriptional and functional level in comparison with naive control cells^4,5,16^ (Extended Data Fig. 4e–k). We confirmed and extended these findings by measuring several aspects of lipid trafficking and utilization upon stimulating BMDMs with LPS/IFNγ. Activated macrophages increased LD accumulation (Fig. 1c and Extended Data Figs. 2a and 4f), triglyceride (TG) levels (Extended Data Fig. 4g) and uptake of fatty acids, which were subsequently incorporated into TGs (Extended Data Fig. 4h,i). In contrast, fatty acid oxidation in mitochondria (Extended Data Fig. 4e,j) or peroxisomes^16^ (Extended Data Fig. 4e) was reduced. Also, mitochondria-driven de novo synthesis of fatty acids from ^13^C-glucose (Extended Data Fig. 4k) was restricted in these cells. Clearly, inflammatory macrophages rewire their cellular fatty acid flow into LDs, thus contributing to LD accumulation.

**Fig. 3:**
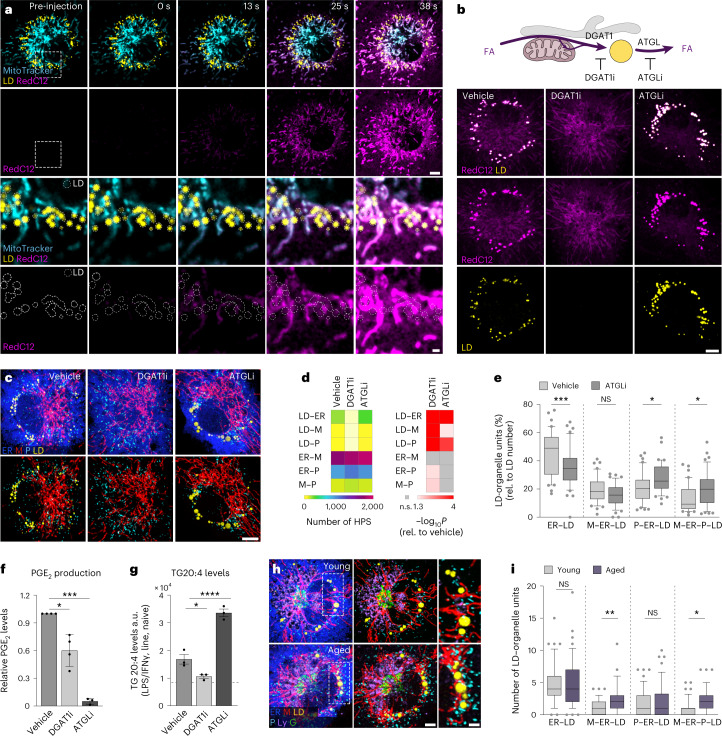
M–ER–P–LD organelles form a fatty acid trafficking unit contributing to and sensing fatty-acid flux in and out of LDs. **a**, Live cell imaging showing the flow of acutely injected fluorescently labelled fatty acids (RedC12, magenta) between M–ER–LD organelles in LPS/IFNγ-activated macrophages (24 h). Bottom: the dotted lines indicate LDs. The images represent *n* = 3 biological replicates. Scale bars, 5 µm (top) and 1 µm (bottom). **b**, RedC12 distribution (24 h pulse) inside and outside of LDs (Lipidspot610) of LPS/IFNγ-treated BMDMs after DGAT1 or ATGL inhibitor (DGAT1i or ATGLi) treatment. FA, fatty acids. The images represent *n* = 3 biological replicates. Scale bar, 5 µm. **c**–**e**, Images (**c**) and quantification of pairwise organelle HPS (**d**) and M–ER–P–LD units (**e**) in LPS/IFNγ-activated BMDMs (24 h) after DGAT1i (**c**,**d**) or ATGLi (**c**–**e**) treatment. Scale bar, 5 µm (**c**). The data represent *N* = 41 (vehicle), *N* = 42 (DGAT1i) and *N* = 41 (ATGLi) cells from *n* = 3 independent experiments. *P* values were obtained using one-way ANOVA with Dunnett’s post hoc test (**d**) or one-way ANOVA with Sidak’s post hoc test (**e**). **f**,**g**, PGE_2_ production (**f**) and TG arachidonic acid content (**g**) of LPS/IFNγ-activated BMDMs (24 h) after DGAT1i or ATGLi treatment. The data represent *n* = 4 (**f**, vehicle, DGAT1i) and *n* = 3 (**f**, ATGLi; **g**) independent experiments. Data are mean ± s.d. (**f**) and mean ± s.e.m. (**g**). *P* values were calculated using two-tailed, one-sample *t*-test (**f**) or one-way ANOVA with Sidak’s post hoc test (**g**). **h**,**i**, Images (**h**) and quantification (**i**) of M–ER–P–LD units in LPS/IFNγ-treated (24 h) peritoneal macrophages isolated from young and aged mice. Scale bars, 3 µm (left) and 2 µm (right) (**h**). Data represent *N* = 46 (young) and *N* = 47 (aged) cells from *n* = 3 biological replicates. *P* values were obtained using Kruskal–Wallis with Dunnett’s post hoc test. Box plots (**e**,**i**) are as in Fig. 2e. Images represent single confocal *z*-planes (**a**,**b**) or maximum intensity projections (**c**,**h**). The numerical *P* values are available in Supplementary Table 4. *P ≤ 0.05, **P ≤ 0.01, ***P ≤ 0.001, ****P ≤ 0.0001. Source numerical data are available in Source data. Source data

**Extended Data Fig. 5:**
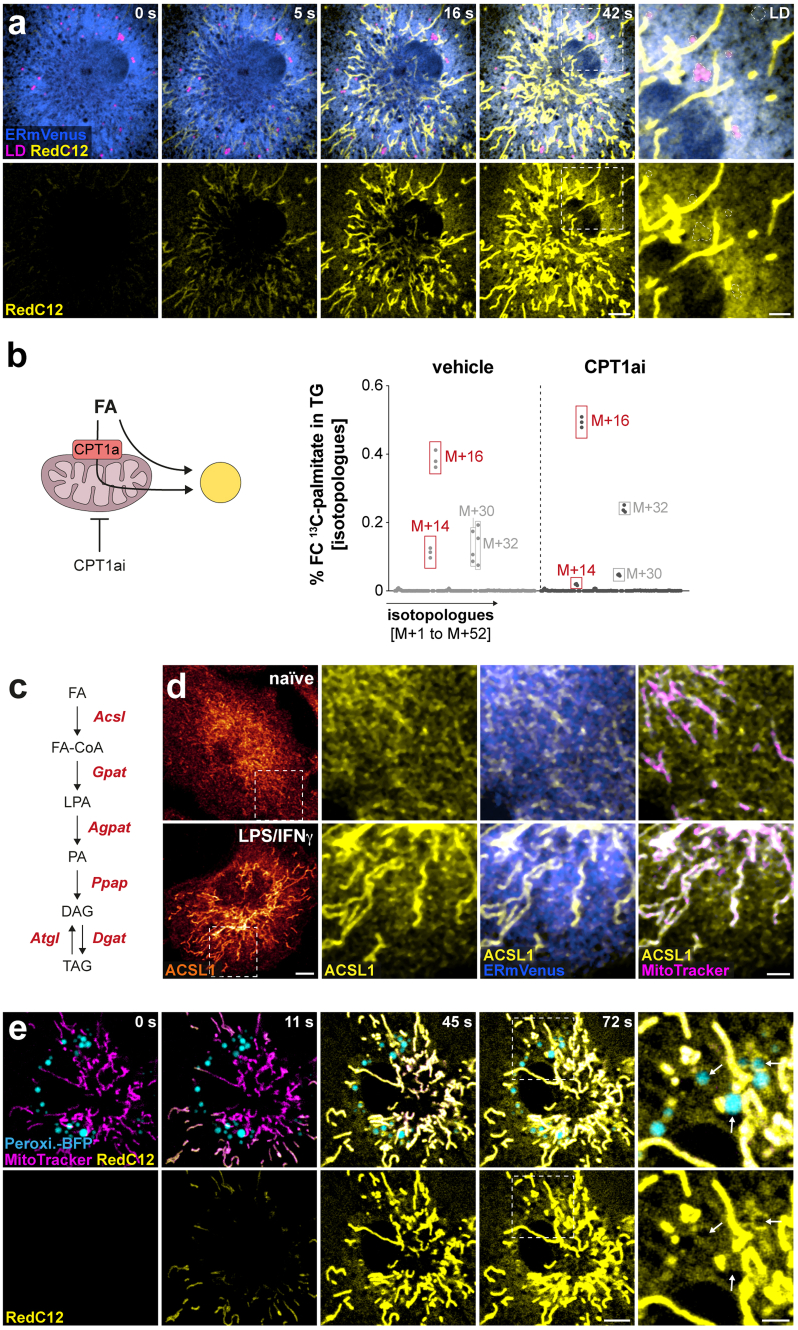
LD fuelling occurs along mitochondria and the ER. **a** Live cell confocal imaging of inflammatory macrophages (LPS/IFN-γ 24 h) showing the flow of acutely injected RedC12 (yellow) in ER (blue) and LDs (Bodipy^493/502^, magenta). Dotted circles in magnifications indicate LDs. Images are single confocal z-planes and represent n = 3 experimental replicates. Scale bars: 5 µm, 2 µm (magnification). **b** Metabolic tracing analysis showing ^13^C-palmitate isotopologues identified in TGs of activated BMDMs (LPS/IFN-γ 24 h) in the presence or absence of the CPT1a inhibitor etomoxir (CPT1ai). Red boxes indicate M+16 and M+14, grey boxes M+30 and M+32 isotopologues. Data represent n = 3 biologically independent replicates. **c** Scheme depicting metabolites (black) and enzymes (red) of the LD biogenesis pathway. FA, fatty acid; FA-CoA, fatty acyl-CoA; PA, phosphatic acid, LPA, lysophosphatidic acid, DG, diacylglycerol; TG, triacylglycerol. **d** Immunofluorescence images showing the cellular distribution of ACSL1 in naïve and LPS/IFN-γ-treated BMDMs (24 h). Fluorescence intensities are displayed as glow heatmap (left). Magnifications show the localization of ACSL1 (yellow) to ER (blue) and mitochondria (magenta). Images show a single confocal z-plane and are representative of n = 3 biological replicates. Scale bars: 5 µm, 2 µm (magnification). **e** Live cell confocal imaging of inflammatory macrophages (LPS/IFN-γ 24 h) showing the localization of acutely injected RedC12 (yellow) to mitochondria (magenta) and peroxisomes (cyan). Arrows in magnifications indicate peroxisomes. Images are single confocal z-planes and are representative of n = 3 experimental repeats. Scale bars: 5 µm, 2 µm (magnification). Source numerical data are available in source data. Source data

Based on our identification of multi-organellar clusters, we hypothesized that the M–ER–P–LD unit could support the inflammation-driven flow of intracellular fatty acids and LD accumulation. To address whether M–ER–P–LD organelles act together as functional unit, we monitored LD fuelling by pulsing the fluorescently labelled fatty acid RedC12 into 24 h-activated BMDMs^17^. Time-lapse imaging revealed that the RedC12 trace co-localized primarily with mitochondria (Fig. 3a). Originating from peripheral mitochondria, the signal rapidly spread over the next 38 s across the mitochondrial network towards the cellular midzone, where RedC12 reached the ER and eventually accumulated in LDs (Fig. 3a and Extended Data Fig. 5a). Stable isotope tracing of ^13^C-palmitate showed that the ingested fatty acids were predominantly shuttled into TGs in a non-metabolized state (isotopologue M + 16), with only a small contribution of metabolized two-carbon-shortened fatty acids (isotopologue M + 14) from mitochondria^18^ (Extended Data Fig. 5b). Thus, our results suggest that inflammatory macrophages use mitochondria and the ER as shuttling systems to transfer primarily non-metabolized fatty acids into LDs. Fatty acid trafficking along both organelles is likely mediated via previously described mitochondria- or ER-localized fatty acid activation enzymes, for example, ACSL1 or ACSL4, whose expression is also upregulated upon inflammation^19–21^ (Extended Data Figs. 4e and 5d) and, thus, can ultimately supply fatty acids for TG synthesis and LD formation^13^. In contrast to the three M–ER–LD organelles, peroxisomes incorporated only minor amounts of RedC12 (Extended Data Fig. 5e), which agrees with their reported inactivation upon LPS/IFNγ^16^ and their predominant role in very-long- rather than long-chain fatty acid metabolism^22^.

### The M–ER–P–LD unit controls inflammatory lipid trafficking and PGE_2_ production

**Extended Data Fig. 6:**
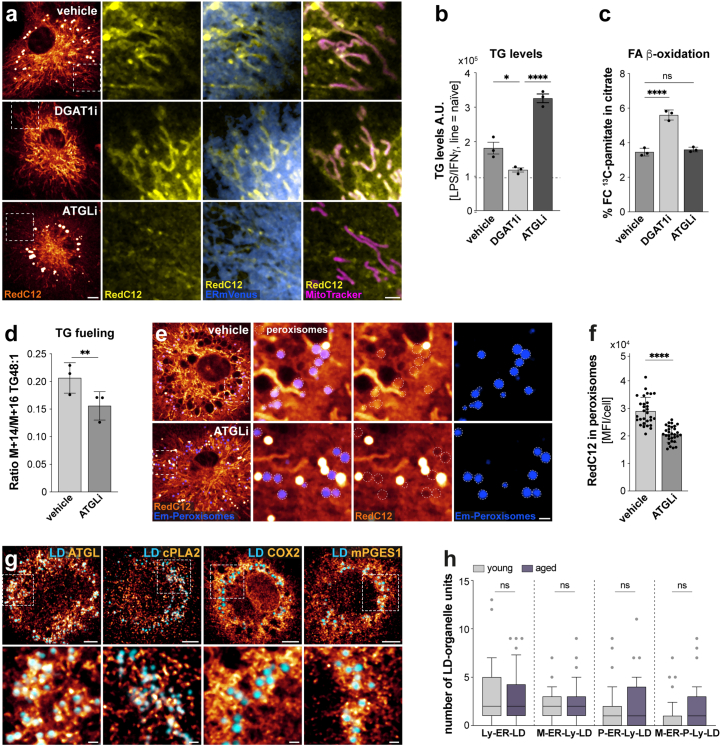
Inhibition of LD biogenesis or lipolysis alters inflammatory fatty acid flow between organelles. **a** Images showing the cellular distribution of RedC12 in LPS/IFN-γ-activated BMDMs (24 h) upon DGAT1i or ATGLi. RedC12 fluorescence intensities are displayed as glow heatmap (left). Magnifications show RedC12 (yellow) localizing to ER (blue) and mitochondria (magenta). Data are representative of n = 3 experimental repeats. Scale bars: 5 µm, 2 µm (magnification). **b** Relative TG content of LPS/IFN-γ-activated BMDMs (24 h) upon DGAT1i or ATGLi. Dotted line indicates TG levels of naïve macrophages. Data represent n = 3 biological repeats. *P* values were calculated using one-way ANOVA with Tukey’s post-hoc test. **c,d** ^13^C-palmitate metabolic tracing analysis showing the fatty acid oxidation (**c**) and the M+14 to M+16 ratio of ^13^C-palmitate isotopologues in TGs (**d**) of LPS/IFN-γ-activated BMDMs upon DGAT1i (**c**) or ATGLi (**c,d**). Data represent n = 3 biological repeats. *P* values were calculated using one-way ANOVA with Dunnett’s post-hoc (**c**) and two-tailed, paired *t* tests (**d**). **e,f** Images (**e**) and quantification (**f**) of RedC12 fluorescence in peroxisomes of LPS/IFN-γ-treated macrophages (24 h) with or without ATGLi. Scale bars: 5 µm, 2 µm (magnification). Data represent N = 40;41 (vehicle; ATGLi) cells from n = 3 biological replicates. Dots represent the MFI of individual cells. *P* value was calculated using two-tailed, unpaired *t*-test (**f**). **g** Images showing the cellular distribution of enzymes mediating TG and phospholipid arachidonic acid release (ATGL, cPLA2; left) and PGE_2_ biogenesis (Cox2, mPGES1, right) in LPS/IFN-γ-treated BMDMs representative of n = 3 biological replicates. Magnifications show their localization relative to LDs. Scale Bars: 5 µm (top), 1 µm (bottom). **h** Lysosome-containing LD-organelle units in young and aged LPS/IFN-γ-treated peritoneal macrophages. Boxes represent 25^th^ to 75^th^ percentiles, whiskers 10^th^ and 90^th^ percentiles. Dots are outliers, the median is shown as a line. Data represents N = 46;47 (young; aged) cells from n = 3 biological replicates. *P* values were obtained using Kruskal-Wallis with Dunnett’s post-hoc test. All images represent single confocal z-planes (**a,e,g**). The bars show the mean ± SEM (**b**), all other bars show mean ± SD (**c, d, f**). Numerical *P* values are available in Supplementary Information Table 4 (*P ≤ 0.05, **P ≤ 0.01, ***P ≤ 0.001, ****P ≤ 0.0001, not significant (ns) *P* > 0.05). Source numerical data are available in source data. Source data

To understand fatty acid trafficking paths within the M–ER–P–LD unit in more detail, we interfered with fatty acid sequestration and distribution at LDs (Fig. 3b). Pharmacological inhibition of the major TG-synthase DGAT1 (T863, DGAT1i)^23^ prevented TG synthesis and abolished LD formation (Fig. 3b). Consequently, fatty acid traces were not incorporated into LDs and TGs (Fig. 3b and Extended Data Fig. 6a,b) and routed instead back to the ER and mitochondria (Fig. 3b and Extended Data Fig. 6a), where fatty acids were metabolized by β-oxidation (Extended Data Fig. 6c). Conversely, chemical inhibition of the lipase ATGL (atglistatin) prevented LD lipolysis^24^ (Extended Data Fig. 6b) and sequestered RedC12 in expanding LDs (Fig. 3b). As a consequence, fatty acid supply from LDs to the ER and mitochondria (Fig. 3b and Extended Data Fig. 6a) and to a minor extent peroxisomes was limited (Extended Data Fig. 6e,f), and mitochondria-generated M + 14 species in TG were reduced (Extended Data Fig. 6d). Altering LD-flux dynamics had direct effects on LD–organelle interactions and the composition of multi-organelle units (MOUs) (Fig. 3c–e). Notably, peroxisomes reacted strongly to changes in LD dynamics and rearranged their interaction profile (Fig. 3d,e). Thus, our data show that inflammatory macrophages can arrange M–ER–P–LD organelles as a functional unit for the sensing and trafficking of fatty acids into and out of LDs.

Macrophages have a pivotal role as a primary source of the lipid mediator prostaglandin E_2_ (PGE_2_), known for its crucial modulatory functions in inflammation and immune responses^25^. Building on previous work^6,24^, we confirm the essential role of LD flux dynamics in PGE_2_ synthesis by inflammatory macrophages (Fig. 3f). Specifically, LDs serve as reservoirs for the release of the PGE_2_ precursor arachidonic acid (C20:4 fatty acid), thereby regulating access to arachidonic acid by the PGE_2_-synthesizing enzymes COX2 and mPGES1^6^ (Fig. 3f,g). The expression of these enzymes is upregulated upon inflammatory macrophage activation^26^ and results in a subcellular concentration near LDs (Extended Data Fig. 6g). In light of our findings on M–ER–P–LD-controlled fatty acid trafficking, we sought to investigate the functional role of this MOU for PGE_2_ synthesis. Initial indications of M–ER–P–LD clusters participating in this process emerged during experiments conducted with cells from aged mice, as excessive PGE_2_ production is a well-established condition in aged macrophages^27^. Notably, OrgaPlex analysis of aged, LPS/IFNγ-activated peritoneal macrophages revealed an increased prevalence of M–ER–LD and M–ER–P–LD clusters compared with cells from younger animals (Fig. 3h,i and Extended Data Fig. 6h). This provided a first link between changes in M–ER–P–LD clustering and pathophysiological states of aberrant LD metabolism.

### MIGA2 promotes the formation of inflammatory M–ER–LD clusters

**Fig. 4:**
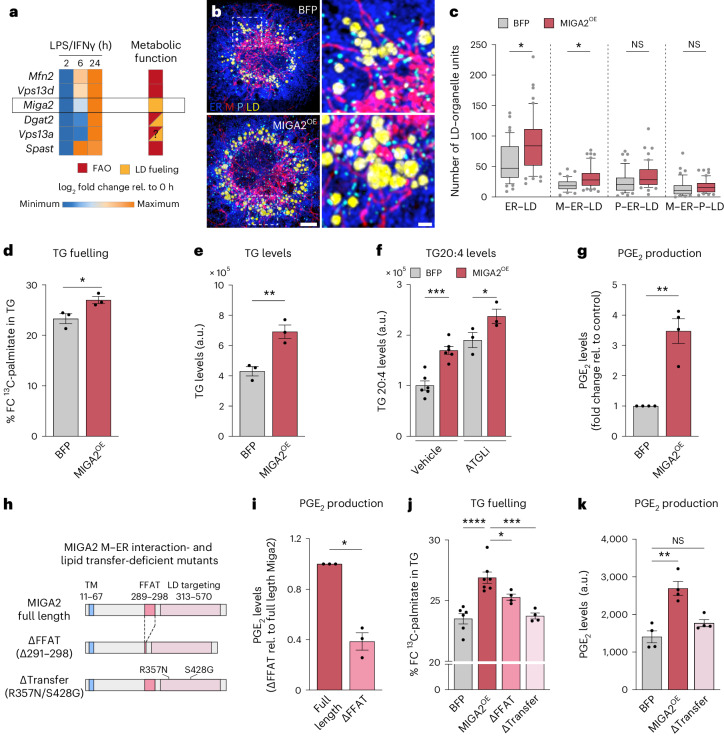
MIGA2 is a M–ER–LD tether regulating LD fuelling of fatty acids and macrophage PGE_2_ production. **a**, Left: LD–organelle tethers identified by RNA-seq with upregulated gene expression during 6–24 h of LPS/IFNγ-treatment. Right: metabolic functions of tethers are indicated by a colour code. FAO, fatty acid oxidation. **b**,**c**, Images (**b**) and quantification (**c**) of M–ER–P–LD units in LPS/IFNγ-activated (24 h) MIGA2-expressing BMDMs. The images are maximum intensity projections. Scale bars, 5 µm and 1 µm (magnification) (**b**). Box plots are as in Fig. 2e. Data represent *N* = 46 (BFP) and *N* = 50 (MIGA2) cells from *n* = 3 biological replicates. *P* values were obtained using a one-way ANOVA with Sidak’s post hoc test (**c**). **d**–**f**, TG analysis showing ^13^C-palmitate fuelling in TG (**d**), TG levels (**e**) and TG arachidonic acid content and release (**f**) of LPS/IFNγ-activated BMDMs (24 h) expressing BFP or MIGA2. The data stem from *n* = 3 (**d**,**e**) or *n* = 6 (**f**, vehicle) biological repeats. *P* values were calculated using two-tailed, unpaired *t*-tests (**d**,**e**) or one-way ANOVA with Sidak’s post hoc test (**f**). **g**, PGE_2_ levels of LPS/IFNγ-treated (24 h), in MIGA2- relative to BFP-expressing BMDMs, representing *n* = 4 biological replicates. *P* value was generated using a two-tailed, one-sample *t*-test. **h**, Scheme representing MIGA2 and its M–ER tethering-deficient MIGA2^ΔFFAT^ and lipid transfer-deficient MIGA2^Δtransfer^ mutants. **i**, PGE_2_ levels produced by MIGA2- or MIGA2^ΔFFAT^-expressing BMDMs (LPS/IFNγ 24 h), representing *n* = 3 biological repeats. *P* value was generated using a two-tailed, one-sample *t*-test. **j**, ^13^C-palmitate fuelling in TG in LPS/IFNγ-treated BMDMs (24 h) expressing BFP, full-length MIGA2 or MIGA2^ΔFFAT^ and MIGA2^Δtransfer^ mutants. FC, fractional concentration. Data represent *n* = 6 (BFP), *n* = 7 (MIGA2) and *n* = 4 (MIGA2^ΔFFAT^, MIGA2^Δtransfer^) independent repeats. *P* values were obtained using a one-way ANOVA with Dunnett’s post hoc test. **k**, PGE_2_ levels of BFP, MIGA2- or MIGA2^Δtransfer^-expressing BMDMs (LPS/IFNγ 24 h), representing *n* = 4 biological replicates. *P* value was generated using Kruskal–Wallis and Dunn’s post hoc test. Data are mean ± s.e.m. (**d**–**g**,**i**–**k**). Numerical *P* values are available in Supplementary Table 4. *P ≤ 0.05, **P ≤ 0.01, ***P ≤ 0.001, ****P ≤ 0.0001. Source numerical data are available in Source data. Source data

**Extended Data Fig. 7:**
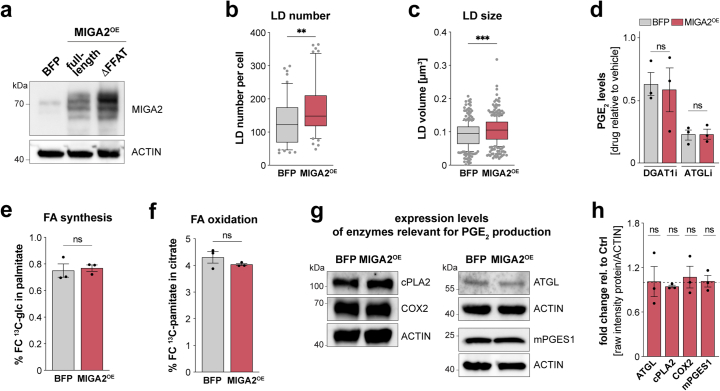
MIGA2 increases LD mass but does not impair mitochondrial fatty acid synthesis or oxidation. **a** Western Blot image of MIGA2 and its M-ER tethering-deficient mutant MIGA2^DFFAT^. Data is representative of n = 2 experimental repeats **b,c** LD number (**b**) and volume (**c**) in BFP or MIGA2 expressing cells. Data represent N = 51;55 (BFP; MIGA2) cells from n = 3 biological repeats. *P* values were obtained using two-tailed Mann Whitney tests. Boxes represent 25^th^ to 75^th^ percentiles, whiskers 10^th^ and 90^th^ percentiles. Dots are outliers, the median is shown as a line. Data represent N = 46;50 (BFP; MIGA2) cells from n = 3 biological replicates. **d** PGE_2_ levels of BFP or MIGA2-expressing LPS/IFN-γ-activated (24 h) BMDMs upon DGAT1i or ATGLi treatment. Data represent n = 3 biological replicates. *P* values were generated using one-way ANOVA with Sidak’s post-hoc test. **e,f** Metabolic tracing analysis showing FA synthesis from ^13^C-glucose (**e**) and FA oxidation from ^13^C-palmitate (**f**) in BMDMs expressing BFP or MIGA2 (LPS/IFN-γ 24 h). Data show n = 3 biological repeats. *P* values were calculated using two-tailed, unpaired *t* tests. **g,h** Representative Western Blot images (**g**) and quantification (**h**) showing protein levels of enzymes involved in FA release (ATGL, cPLA2) and PGE_2_ biogenesis (COX2, mPGES1) in LPS/IFN-γ-activated (24 h) BMDMs upon MIGA2 overexpression. Data represent n = 3 biological replicates. All bars show mean ± SEM (**d-f**). *P* values were calculated using two-tailed, one-sample *t* tests. Numerical *P* values are available in Supplementary Information Table 4 (*P ≤ 0.05, **P ≤ 0.01, ***P ≤ 0.001, ****P ≤ 0.0001, not significant (ns) *P* > 0.05). Source numerical data and unprocessed blots are available in source data. Source data

To verify the functional role of M–ER–P–LD units for PGE_2_ production, we next searched for molecular targets that would potentially allow the modulation of MOU assembly and function. First, we performed time-resolved RNA sequencing (RNA-seq) of control and LPS/IFNγ-stimulated BMDMs. Among the differentially expressed genes, we specifically focused on known LD-tether proteins with an upregulated expression between 6–24 h after activation, thus correlating with M–ER–LD, P–ER–LD and M–ER–P–LD cluster formation (Fig. 2d,e). Six candidate tether proteins were annotated together with their reported function for LD metabolism^28–35^ (Fig. 4a). *Miga2*, a recently discovered tether facilitating mitochondria interactions with the ER and LDs in adipocytes^31,36^, was identified as the only exclusive LD-fuelling regulator (Fig. 4a). While we did not manage to knockdown or deplete MIGA2 in primary BMDMs, we ectopically expressed MIGA2 to gain insight into its role for MOU assembly (Extended Data Fig. 7a). In line with its reported LD-fuelling function, ectopic expression of MIGA2 enhanced LD number and size (Extended Data Fig. 7b,c) and induced a selective remodelling of LD-containing MOUs, including the formation of ER–LD and M–ER–LD clusters (Fig. 4b,c). Moreover, metabolic measurements determined that MIGA2 enhanced the incorporation of taken-up exogenous fatty acids into TGs (Fig. 4d) and increased total TG levels (Fig. 4e), as well as their storage and release of arachidonic acid (Fig. 4f). Ultimately, these changes in lipid flow were accompanied by a threefold increase in LD-controlled PGE_2_ synthesis (Fig. 4g and Extended Data Fig. 7d). Other aspects of PGE_2_-relevant lipid metabolism remained unaffected (Extended Data Fig. 7e–h).

### MIGA2 amplifies inflammatory lipid trafficking and PGE_2_ production

**Extended Data Fig. 8:**
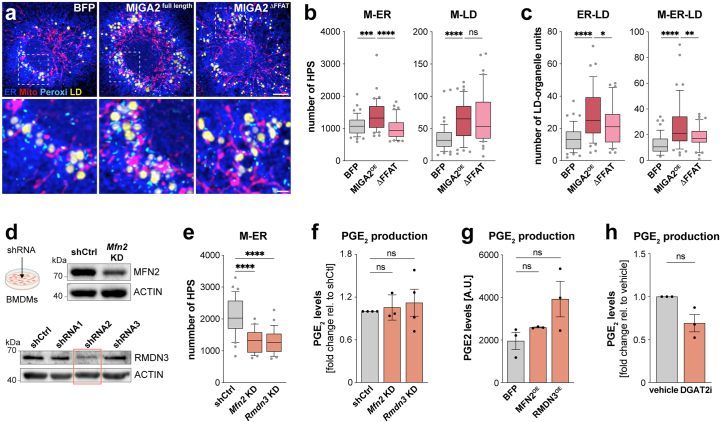
MIGA2 but not other M-ER-LD tethers contribute to macrophage PGE_2_ production. **a** Images depicting M-ER-P-LD units in LPS/IFN-γ-activated BMDMs (24 h) expressing BFP, MIGA2 or MIGA2^DFFAT^. Images represent maximum intensity projections of n = 3 biolgocial replicates. Scale bars: 5 µm, 1 µm (magnification). **b,c** Pair-wise M-ER and M-LD high proximity sites (**b**) and MIGA2-regulated LD-units (**c**) in LPS/IFN-γ-treated (24 h) BMDMs expressing BFP, MIGA2 or MIGA2^DFFAT^. Data represent N = 48 (BFP), N = 48 (MIGA2) and N = 40 (MIGA2^DFFAT^) cells from n = 3 biologically independent replicates. Boxes represent 25^th^ to 75^th^ percentiles, whiskers 10^th^ and 90^th^ percentiles. Dots are outliers, the median is shown as a line. *P* values were obtained using one-way ANOVA with Dunnett’s post-hoc test. **d** Western Blot analysis showing knock-down (KD) efficiency of *Mfn2 or Rmdn3* in BMDMs representative of n = 1 experiment. **e** Pairwise ER-mitochondria HPS analysis in *Mfn2* KD*, Rmdn3* KD or control BMDMs. Data represent N = 32;24;29 (shCtrl; *Mfn2* KD; *Rmdn3* KD) cells from n = 3 biological replicates. Boxes represent 25^th^ to 75^th^ percentiles, whiskers 10^th^ and 90^th^ percentiles. Dots are outliers, the median is shown as a line. *P* values were calculated using one-way ANOVA with Dunnett’s post hoc test. **f-h** PGE_2_ production of LPS/IFN-γ-treated BMDMs (24 h) upon *Mfn2* and *Rmdn3* KD (**f**), MFN2 and RMDN3 overexpression (**g**) or DGAT2 inhibition (DGAT2i) (**h**). Data represent n = 4 (**f**, *Rmdn3* KD) and n = 3 (**f**, *Mfn2* KD; **g-h**) biologically independent experiments. *P* values were calculated using two-tailed, one-sample *t* tests (**f,h**) or one-way ANOVA with Dunnett’s post-hoc test (**g**). Bars show mean ± SEM (**f-h**). Numerical *P* values are available in Supplementary Information Table 4 (*P ≤ 0.05, **P ≤ 0.01, ***P ≤ 0.001, ****P ≤ 0.0001, not significant (ns) *P* > 0.05). Source numerical data and unprocessed blots are available in source data. Source data

To further validate the link between MIGA2-driven LD-cluster assembly, TG fuelling and PGE_2_ synthesis, we next sought to manipulate aspects of MIGA2 tethering function. Expressing a MIGA2 mutant with a deleted M–ER interaction domain (MIGA2^ΔFFAT^)^31,36^ (Fig. 4h and Extended Data Fig. 7a) resulted in the anticipated constriction of ER–M interactions (ER-M HPS) induced by full-length MIGA2 (Extended Data Fig. 8a,b). Beyond this, FFAT loss prompted a distinct rewiring of the LD interactome and selectively restricted the ER–LD and M–ER–LD clusters (Extended Data Fig. 8c). By contrast, pairwise LD–M interactions, which can be sustained by other MIGA2 domains^31^, were largely unaffected by the loss of FFAT (Extended Data Fig. 8b). FFAT loss and the resulting reduction of M–ER–LD cluster assembly was sufficient to decrease the levels of PGE_2_ production (Fig. 4i) and TG fuelling (Fig. 4j) relative to MIGA2-expressing cells. Further confirmation for MIGA2’s role in promoting TG fuelling and PGE_2_ production was obtained by the direct mutation of key residues^36^ in the lipid-transfer domain of MIGA2 (Δtransfer)^36,37^ (Fig. 4h). Remarkably, the expression of this transfer mutant almost completely abolished the TG fuelling capacity and PGE_2_ production induced by wild-type MIGA2 (Fig. 4j,k). Thus, our data strongly support a role for MIGA2 in regulating LD cluster formation, LD fuelling and PGE_2_ production. Efforts to manipulate other M–ER–LD-relevant protein tethers or regulators, including MFN2 (M–ER and M–LD tether)^28,38^, RMDN3 (M–ER or M–ER–LD tether)^39,40^ or to functionally interfere with DGAT2 (ER–LD, M–ER–LD localization)^33^, did not result in significantly altered PGE_2_ synthesis (Extended Data Fig. 8d–h). Together, our results support the notion that the upregulated MIGA2 expression of late inflammatory macrophages contributes to the formation of M–ER–LD units to facilitate exogenous lipid flow into LDs and PGE_2_ production.

### The mitochondrial fission regulator DRP1 controls M–ER–P–LD cluster assembly and PGE_2_ production

**Fig. 5:**
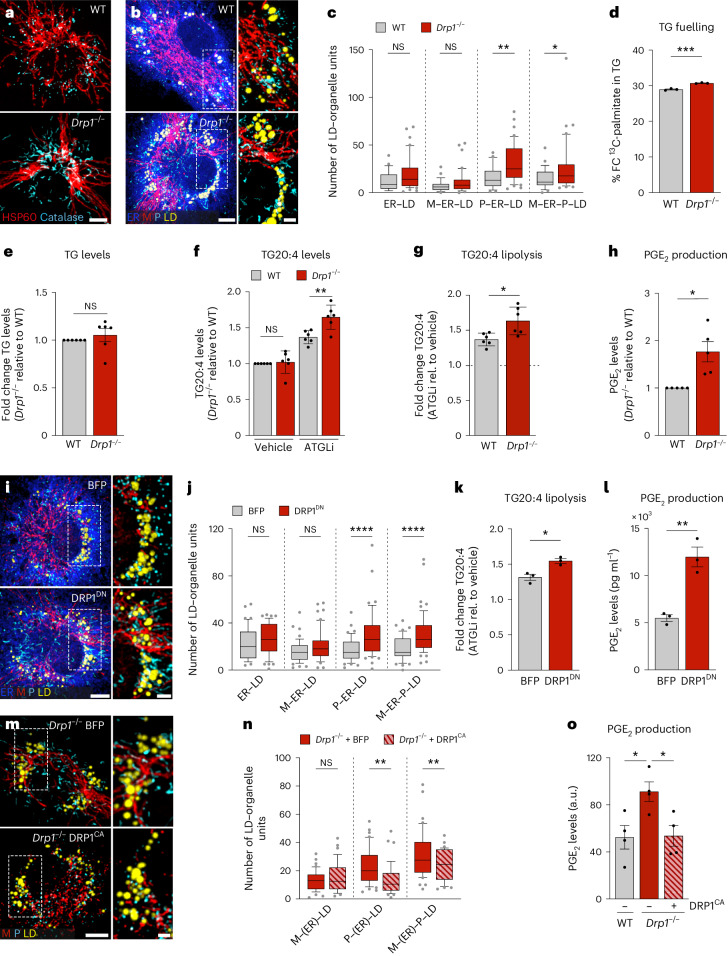
DRP1 regulates M–ER–P–LD cluster formation and PGE_2_ production. **a**, Images showing mitochondrial (red) and peroxisomal (cyan) morphologies in inflammatory *Drp1*^*−/−*^ or wild-type (WT) BMDMs representing *n* = 3 biological replicates. **b**,**c**, Images (**b**) and quantification (**c**) of M–ER–P–LD clusters in LPS/IFNγ-activated *Drp1*^*−/−*^ or wild-type BMDMs. The data represent *N* = 28 (WT) and *N* = 42 (*Drp1*^*−/−*^) cells from *n* = 3 biological replicates. *P* values were obtained using one-way ANOVA with Sidak’s post hoc test. **d**–**g**, TG analysis showing ^13^C-palmitate fuelling in TG (**d**), TG levels (**e**), TG arachidonic acid content (**f**) and release (**f**,**g**) in LPS/IFNγ-treated wild-type and *Drp1*^*−/−*^ BMDMs (24 h) representative of *n* = 3 (**d**) or *n* = 6 (**e**–**g**) biological replicates. *P* values were obtained using a two-tailed, unpaired *t*-test (**d**,**g**), two-tailed, one-sample *t*-test (**e**) or one-way ANOVA with Sidak’s post hoc test (**f**). **h**, PGE_2_ production of LPS/IFNγ-activated wild-type or *Drp1*^*−/−*^ macrophages (24 h) representing *n* = 5 biological repeats. *P* value was obtained using a two-tailed, one-sample *t*-test. **i**,**j**, Images (**i**) and quantification (**j**) of M–ER–P–LD clusters in LPS/IFNγ-activated DRP1^DN^ expressing BMDMs. Data represent *N* = 48 (BFP) and *N* = 55 (DRP1^DN^) cells from *n* = 3 biological repeats. *P* values were obtained using a one-way ANOVA with Sidak’s post hoc test (**j**). **k**,**l**, TG arachidonic acid release (**k**) and PGE_2_ production (**l**) of BFP and DRP1^DN^ expressing LPS/IFNγ-treated BMDMs (24 h). Data show *n* = 3 independent measurements. *P* values were obtained using two-tailed, unpaired *t*-tests (**k**,**l**). **m**,**n**, Images (**m**) and quantification (**n**) of M–ER–P–LD clusters in LPS/IFNγ-activated DRP1^CA^- or BFP-expressing *Drp1*^*−/−*^ BMDMs. Data represent *N* = 42 (BFP) and *N* = 38 (DRP1^DN^) cells from *n* = 3 biological replicates. *P* values were obtained using one-way ANOVA with Sidak’s post hoc test (**n**). **o**, PGE_2_ production of LPS/IFNγ-treated wild-type BMDMs expressing BFP and *Drp1*^*−/−*^ BMDMs expressing BFP or DRP1^CA^. Data show *n* = 4 biological replicates. *P* values were obtained using one-way ANOVA with Dunnett’s post hoc test. All images are maximum intensity projections. Scale bars, 5 µm and 2 µm (magnifications) (**a**,**b**,**i**,**m**). Box plots (**c**,**j**,**n**) are as in Fig. 2e. Data are mean ± s.e.m. (**d**,**e**,**h**,**k**,**l**) or mean ± s.d. (**f**,**g**). Numerical *P* values are available in Supplementary Table 4. *P ≤ 0.05, **P ≤ 0.01, ***P ≤ 0.001, ****P ≤ 0.0001. Source numerical data are available in Source data. Source data

**Extended Data Fig. 9:**
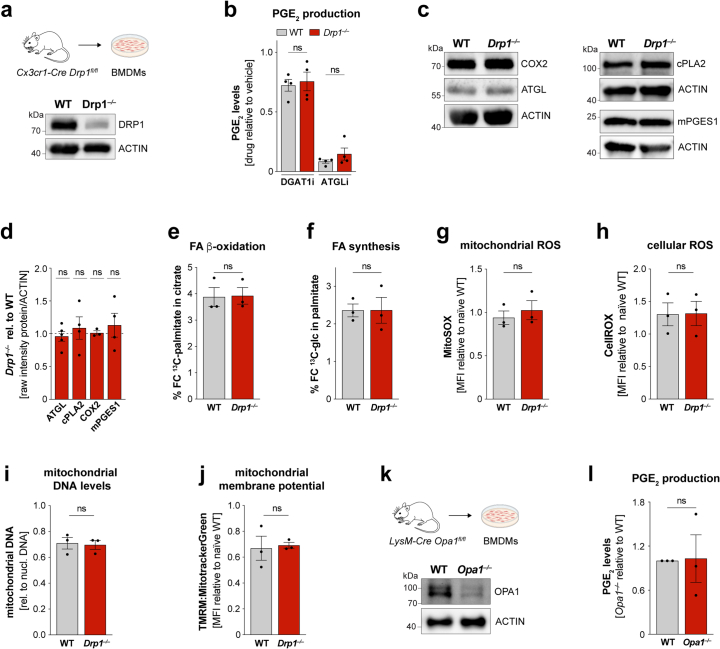
DRP1-regulated PGE_2_ production is not driven by changes in key mitochondrial functions. **a** Western Blot analysis showing the KO efficiency of *Drp1*^*−/−*^ macrophages obtained from *Cx3cr1-Cre Drp1*^*fl/fl*^ mice representative of n = 3 biological replicates. **b** PGE_2_ levels of LPS/IFN-γ-activated (24 h) WT and *Drp1*^*−/−*^ BMDMs treated with DGAT1i or ATGLi representing n = 4 biological replicates. *P* values were generated using one-way ANOVA with Sidak’s post-hoc test. **c,d** Western Blot images (**c**) and quantification (**d**) showing protein levels of enzymes involved in FA release (ATGL, cPLA2) and PGE_2_ biogenesis (COX2, mPGES1) in LPS/IFN-γ-treated WT and *Drp1*^*−/−*^ BMDMs (24 h). Data represent n = 3 (COX2) and n = 4 (cPLA2, mPGES1) and n = 5 (ATGL) biological replicates (**c,d**). *P* values were obtained using two-tailed, one-sample t tests (**d**). **e,f** Metabolic tracing analysis of LPS/IFN-γ-activated WT and *Drp1*^*−/−*^ BMDMs showing ^13^C-palmitate fueling in citrate (FA oxidation) (**e**) and ^13^C-glucose fueling into palmitate (FA synthesis) (**f**) from n = 3 biologically independent replicates. *P* values were obtained using two-way, unpaired *t* tests. **g,h** Mitochondrial (**g**) and cellular ROS (**h**) measurements in LPS/IFN-γ-treated (24 h) WT and *Drp1*^*−/−*^ BMDMs obtained by flow cytometry in n = 3 biologically independent experiments. *P* values were calculated using two-way, unpaired *t* tests. **i** Quantification of mitochondrial DNA content in LPS/IFN-γ-activated WT and *Drp1*^*−/−*^ BMDMs by qRT-PCR from n = 3 biological replicates. *P* value was obtained using two-way, unpaired *t* test (ns *P* > 0.05). **j** Measurement of mitochondrial membrane potential (TMRM) relative to MitoTrackerGreen in LPS/IFN-γ-treated WT and *Drp1*^*−/−*^ BMDMs. Data represent n = 3 biological replicates. *P* value was obtained using two-way, unpaired *t* test. **k** Western Blot analysis showing the knock-out efficiency of *Opa1*^*−/−*^ macrophages compared to WT BMDMs representative of n = 3 biological replicates **l** PGE_2_ levels produced by LPS/IFN-γ-activated WT or *Opa1*^*−/−*^ BMDMs (24 h). Data represent n = 3 biologically independent replicates. *P* value was calculated using two-tailed, one-sample *t* test. All bars show mean ± SEM (**b,d-j,l**). Numerical *P* values are available in Supplementary Information Table 4 (*P ≤ 0.05, **P ≤ 0.01, ***P ≤ 0.001, ****P ≤ 0.0001, not significant (ns) *P* > 0.05). Source numerical data and unprocessed blots are available in source data. Source data

As identified by OrgaPlexing, LD accumulation synchronized with mitochondrial tubulation at 16–24 h and the selective inactivation of DRP1 after macrophage activation (Fig. 1c and Extended Data Fig. 2c–i). Although DRP1 is mostly known as major mitochondrial fission regulator, it also controls peroxisomal fission and has been linked to ER–M interactions and lipid trafficking^41–43^, thus making DRP1 a potential candidate for modulating M–ER–P–LD units and PGE_2_ production. As expected, *Drp1*^*−/–*^ BMDMs (Extended Data Fig. 9a) showed elongated mitochondria and peroxisomes (Fig. 5a). In addition, *Drp1* deficiency induced the assembly of P–ER–LD and M–ER–P–LD clusters (Fig. 5b,c). The increased presence of peroxisome-containing MOUs was accompanied by mild increases in LD fuelling (Fig. 5d), which likely occurs along ER–M interactions of the MOU^41,43^. Although the elevated fuelling did not result in upregulated TG and TG-stored arachidonic acid levels (Fig. 5d–f), *Drp1*^*−/−*^ BMDMs showed a substantial increase in TG-stored arachidonic acid upon ATGL inhibition (Fig. 5f,g). Thus, *Drp1*^*−/−*^ BMDMs enhance the flux of fatty acids into and arachidonic acid release from TGs, consequently resulting in higher PGE_2_ production (Fig. 5h and Extended Data Fig. 9b). Expression of the dominant-negative fission mutant DRP1^K38A^ (DRP1^DN^)^44^ mimicked all key effects of *Drp1*^*−/−*^ BMDMs, including MOU formation, TG-stored arachidonic acid release and PGE_2_ production (Fig. 5i–l). Conversely, expressing a catalytically active DRP1 mutant (DRP1^CA^)^45^ in *Drp1*^*−/−*^ BMDMs counteracted both the formation of peroxisome containing MOUs and PGE_2_ production (Fig. 5m–o). Together, these data strongly suggest DRP1 as modulator of lipid trafficking and PGE_2_ production in inflammatory macrophages. The DRP1-controlled increase in PGE_2_ levels could neither be attributed to altered expression of key PGE_2_-producing enzymes (Extended Data Fig. 9c,d) nor be related to changes in key mitochondrial functions (Extended Data Fig. 9e–j). As PGE_2_ levels were unchanged in BMDMs depleted of MFN2 or OPA1, we also concluded that mitochondrial fusion was per se not essential for PGE_2_ synthesis (Extended Data Fig. 8f,g and Extended Data Fig. 9k,l). Instead, our LD-interactome analysis of *Drp1*^*−/−*^ BMDMs pointed towards peroxisomes as additional regulator of PGE_2_ production (Fig. 5b,c).

### DRP1-regulated P–LD tethering controls inflammatory PGE_2_ production

**Fig. 6:**
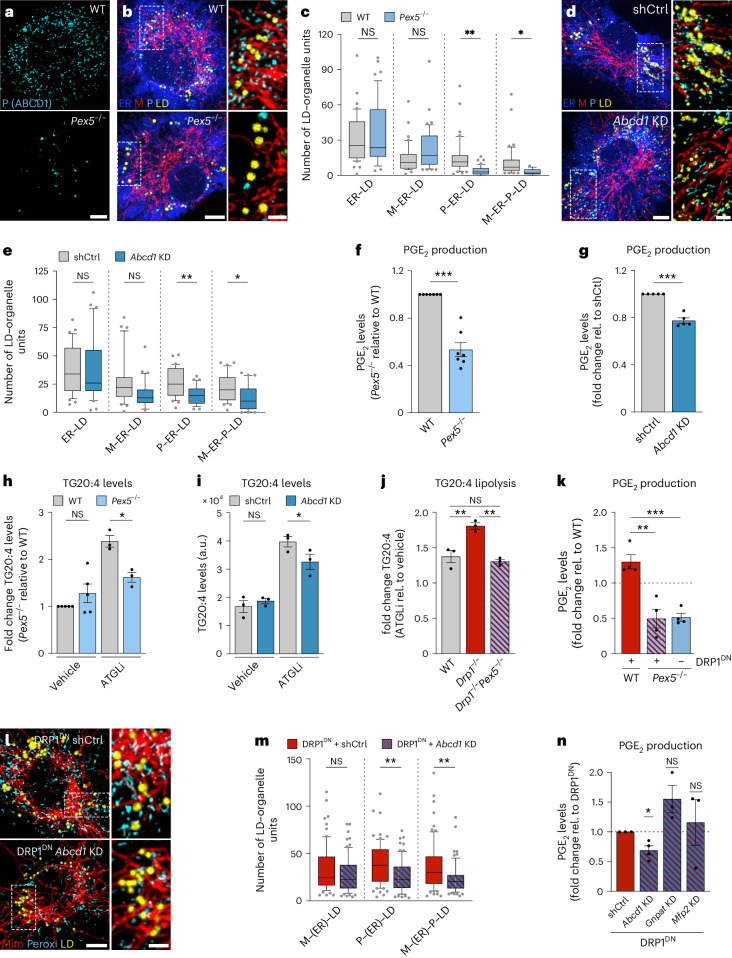
Peroxisomes control the release of LD-stored arachidonic acid and drive PGE_2_ production in Drp1 inhibited cells. **a**, Images showing peroxisomes in wild-type or *Pex5*^*−/−*^ BMDMs representing *n* = 3 biological repeats. **b**–**e**, Images (**b**,**d**) and quantification (**c**,**e**) of M–ER–P–LD clusters in LPS/IFNγ-activated BMDMs upon *Pex5*^*−/−*^ (**b**,**c**) or *Abcd1* knockdown (KD) (**d**,**e**) relative (rel.) to controls. shCtrl, scrambled shRNA control. Data from *N* = 42 (WT) and *N* = 44 (*Pex5*^*−/−*^) and *N* = 31 (shCtrl) and *N* = 33 (*Abcd1* KD) cells were pooled from *n* = 3 biological replicates. *P* values were obtained using a one-way ANOVA with Sidak’s post hoc test (**c**,**e**). **f**,**g**, PGE_2_ production of LPS/IFNγ-activated (24 h) *Pex5*^*−/−*^ (**f**) or *Abcd1* KD (**g**) BMDMs relative to controls. Data represent *n* = 7 (**f**) and *n* = 5 (**g**) biologically independent experiments. *P* values were obtained using two-tailed, one-sample *t*-tests. **h**–**j**, Lipidomic analysis showing TG arachidonic acid content (**h**,**i**) and release (**h**–**j**) in LPS/IFNγ-treated *Pex5*^*−/−*^ (**h**), *Abcd1* KD BMDMs (**i**) or *Drp1*^*−/−*^ and *Pex5*^*−/−*^*Drp1*^*−/−*^ (**j**) BMDMs relative to controls. Data represent *n* = 5 (**h**, vehicle) and *n* = 3 (**i**,**j** and **h**, ATGLi) biological replicates. *P* values were obtained using one-way ANOVA with Sidak’s post hoc test (**h**–**j**). **k**, PGE_2_ production of wild-type or *Pex5*^*−/−*^ BMDMs expressing DRP1^DN^ (LPS/IFNγ 24 h) from *n* = 4 biologically independent experiments. *P* values were obtained using a one-way ANOVA with Tukey’s post hoc test. **l**,**m**, Images (**l**) and quantification (**m**) of M–P–LD clusters LPS/IFNγ-activated DRP1^DN^ overexpressing BMDMs upon *Abcd1* KD. Data represent *N* = 54 (shCtrl) and *N* = 54 (*Abcd1* KD) cells from *n* = 4 biological replicates. *P* values were obtained using Kruskal–Wallis with Dunn’s post hoc test. **n**, PGE_2_ production of LPS/IFNγ-activated DRP1^DN^ overexpressing BMDMs upon *Abcd1*, *Gnpat* or *Mfp2* knockdown. Data represent *n* = 4 (*Abcd1* KD) or *n* = 3 (*Gnpat* and *Mfp2* KD) biological replicates. *P* values were obtained using two-tailed, one-sample *t-*tests. All images are maximum intensity projections. Scale bars, 5 µm and 2 µm (magnifications) (**a**,**b**,**d**,**l**). Box plots (**c**,**e**,**m**) are as in Fig. 2e. Data are mean ± s.e.m. (**f**–**k**,**n**). Numerical *P* values are available in Supplementary Table 4. *P ≤ 0.05, **P ≤ 0.01, ***P ≤ 0.001, ****P ≤ 0.0001. Source numerical data are available in Source data. Source data

**Extended Data Fig. 10:**
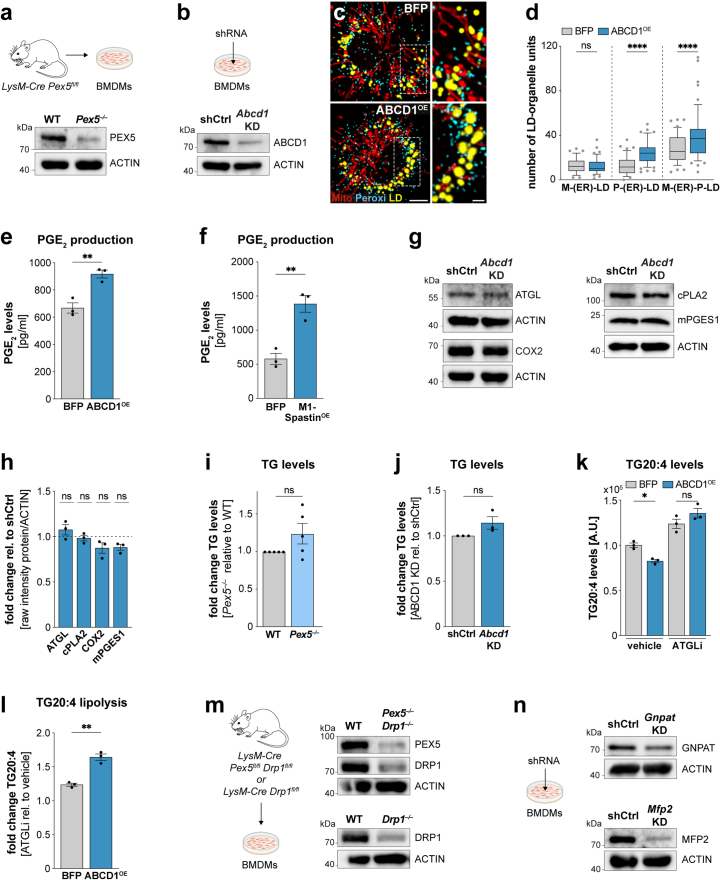
Increased peroxisome-LD tethering drives LD lipolysis and PGE2 production. **a,b** Western Blot analysis showing the knock-out efficiency of *Pex5*^*−/−*^ (**a**) and *Abcd1* KD-efficiency (**b**) relative to control BMDMs representing n = 4;3 (*Pex5*^*−/−*^; *Abcd1* KD) biological repeats. **c,d** Images (**c**) and quantification (**d**) of M-P-LD clusters in LPS/IFN-γ-activated (24 h) BMDMs upon ABCD1 expression. Images represent maximum intensity projections. Scale bars: 5 µm, 2 µm (magnification). Boxes represent 25^th^ to 75^th^ percentiles, whiskers 10^th^ and 90^th^ percentiles. Dots are outliers, the median is shown as a line. Data represent N = 48;53 (BFP; ABCD1) cells from n = 3 biological replicates. *P* values were obtained using one-way ANOVA with Sidak’s post-hoc test. **e-f** PGE_2_ levels of LPS/IFN-γ-treated (24 h) BMDMs expressing ABCD1 (**e**) or M1-Spastin (**f**) representing n = 3 biological replicates. *P* values were obtained using two-tailed, unpaired *t* tests (**e,f**). **g,h** Western Blot images (**g**) and quantification (**h**) showing protein levels of enzymes involved in FA release (ATGL, cPLA2) and PGE_2_ biogenesis (COX2, mPGES1) in LPS/IFN-γ-activated BMDMs upon *Abcd1* KD. Data represent n = 3 biologically independent experiments (**g,h**). *P* values were obtained using two-tailed, one-sample t tests (**h**). **i-j** Triglyceride analysis showing TG levels in LPS/IFN-γ-activated (24 h) *Pex5*^*−/−*^ (**i**) and *Abcd1* KD BMDMs (**j**) relative to controls. Data represent n = 5;3 (**i,j**) biological replicates. *P* values were obtained using two-tailed, one-sample *t* tests. **k,l** TG arachidonic acid content (**k**) and release (**k,l**) of BFP and ABCD1 expressing cells from n = 3 biological replicates. *P* values were obtained using one-way ANOVA with Sidak’s post-hoc (**k**) and two-tailed, unpaired *t* tests (**l**) **m** Western Blot analysis showing the KO efficiency of *Pex5*^*−/−*^ *Drp1*^*−/−*^ and *Drp1*^*−/−*^ BMDMs obtained from *LysM-Cre Drp1*^*fl/f*^*Pex5*^*fl/fl*^ and *LysM-Cre Drp1*^*fl/fl*^ mice, respectively. Data represent n = 3 biological replicates **n** Western Blot analysis showing the efficiency of *Mfp2* and *Gnpat* KD relative to control BMDMs representative of n = 2 independent repeats. All bars show mean ± SEM (**e,f,h-l**). Numerical *P* values are available in Supplementary Information Table 4 (*P ≤ 0.05, **P ≤ 0.01, ***P ≤ 0.001, ****P ≤ 0.0001, not significant (ns) *P* > 0.05). Source numerical data and unprocessed blots are available in source data. Source data

To address the role of peroxisomes for MOU assembly and PGE_2_ synthesis more directly, we used BMDMs genetically deficient for the peroxisomal import protein PEX5^46^ (Extended Data Fig. 10a). *Pex5*^*−/−*^ cells were almost devoid of peroxisomes (Fig. 6a,b), which consequently resulted in substantial reductions of P–ER–LD and M–ER–P–LD clusters (Fig. 6c). In a more refined approach, we manipulated P–LD clustering by modifying a P–LD tether pair, consisting of the LD-localized M1-Spastin, which we also identified as tether candidate from our RNA-seq analysis, and its peroxisomal partner ABCD1 (Figs. 4a and 6d,e and Extended Data Fig. 10b–d). Clearly, depletions of PEX5 or ABCD1 reduced PGE_2_ synthesis, whereas ectopic expression of the P–LD tether molecules ABCD1 or M1-Spastin substantially increased PGE_2_ production (Fig. 6f,g and Extended Data Fig. 10e,f). The observed phenotypes could neither be attributed to altered expression levels of PGE_2_-synthesizing enzymes (Extended Data Fig. 10g,h), nor to changes in total TG or TG-stored arachidonic acid levels (Fig. 6h,i and Extended Data Fig. 10i–k). However, the impairment of peroxisomal function (Fig. 6h) or the disruption of P–LD tethering (Fig. 6i) resulted in a decreased release of arachidonic acid from TGs. Conversely, augmenting P–LD tethering promoted the release of TG-stored arachidonic acid (Extended Data Fig. 10l). Thus, our data support a role of peroxisomes in regulating LD lipolysis, as suggested previously^47–49^.

Remarkably, manipulating peroxisome biology was sufficient to antagonize effects induced upon DRP1 inactivation. Repressing peroxisomal mass using *Drp1*^*−/−*^*Pex5*^*−/−*^ BMDMs (Extended Data Fig. 10m) effectively abrogated the increased release of arachidonic acid from TGs seen in *Drp1*^*−/−*^ BMDMs (Fig. 6j). Consistently, loss of *Pex5* also restricted the enhanced PGE_2_ production induced by DRP1 inactivation using DRP1^DN^ (Fig. 6k). Targeting peroxisomes in a more refined manner by depleting ABCD1 was sufficient to reduce the DRP1^DN^-enforced increase in peroxisome-containing MOUs (Fig. 6l,m) and PGE_2_ production (Fig. 6n). In contrast to the crucial role of P–LD tethering for PGE_2_ production, manipulating peroxisomal functions by depleting MFP2, a key factor for peroxisomal β-oxidation^50^, or GNPAT, a driver of ether-lipid synthesis^51^, did not result in measurable changes in DRP1^DN^-driven PGE_2_ synthesis (Fig. 6n and Extended Data Fig. 10n). Thus, our results demonstrate that inactivation of DRP1 leads to an increased incorporation of peroxisomes into MOUs, likely facilitated by molecular tethers such as ABCD1-M1-Spastin. Formation of these peroxisome-containing MOUs in inflammatory macrophages ultimately amplifies the mobilization of TG-stored arachidonic acid to support PGE_2_ production.

## Discussion

Together, our data define how macrophages rewire their organellar architecture to enable metabolic switches and control macrophage effector functions. We demonstrate that inflammatory macrophages embed LDs in MOUs that regulate inflammatory fatty-acid trafficking. While M–ER–LD clusters enable LD fuelling, the additional recruitment of peroxisomes facilitates LD lipolysis and with it the liberation of arachidonic acid for PGE_2_ production. How these units and their incorporated contact sites control lipid flow remains to be determined in more detail. Beyond controlling fatty acid trafficking efficacy, organelle contacts can alter phospholipid content, membrane curvature, protein activity and organelle function^11,36,37,52^ that may contribute to PGE_2_ production^53,54^. Most importantly, our data show that we are now able to extract MOUs and their molecular regulators from phenotypical OrgaPlexing. This has notable implications, as MOUs start to connect previously disparate parts of the cellular architecture. With increasing numbers of gene mutations found in metabolic and organellar genes and identified organellar defects in pathological states^55–57^, MOUs will likely expand our insight into pathophysiological conditions. Considering solely LD MOUs, this extended organelle regulation unit could provide insights in conditions associated with aberrant lipid and arachidonic acid metabolism, such as aging-induced neuroinflammation^27,58^, bacterial infections^14^ or tumour progression^7,59,60^. As our OrgaPlexing approach requires no genetic manipulations of target cells (Fig. 3h), it is applicable to virtually any cell type, including rare or challenging-to-manipulate primary (immune) cells. This versatility empowers us to investigate organelles as MOUs to further advance our comprehension of organelle physiology and metabolism in both health and disease.

## Methods

Animal breeding and husbandry were performed in accordance with the guidelines provided by the Federation of European Laboratory Animal Science Association and the Regierungsprasidium Freiburg, Germany Az. 35-9185.64/1. The animals were euthanized for tissue removal in compliance to section 4, paragraph 3 of the German Animal Protection Act.

### Mice

The following mouse strains were used: C57BL/6J, C57BL6/J *Cx3cr1*^tm1.1(cre)Jung^ *Dnm1l*^tm1.1Miha^, C57BL6/J *Lyz2*^tm1(cre)Ifo^ *Dnm1l*^tm1.1Miha^, C57BL6/J *Lyz2*^tm1(cre)Ifo^ *Pex5*^tm1(Pec)Baes^, C57BL6/J *Lyz2*^tm1(cre)Ifo^ *Pex5*^tm1(Pec)Baes^ *Dnm1l*^tm1.1Miha^ and C57BL6/J *Lyz2*^tm1(cre)Ifo^ *Opa1*^tm1.1Hise^. C57BL/6J (JAX, no. 000664), *Pex5*^*fl/fl*^ mice (B6J.129-Pex5^*tm1Pec*^/BaesJ; JAX, no. 031665) were purchased from the Jackson Laboratories. *Opa1*^*fl/fl*^ and *Drp1*^*fl/fl*^ mice were a gift from E. Pearce. The mice were housed under specific pathogen-free conditions with 20–24 °C, 45–65% humidity, 14–10 h light–dark cycle and standard chow diet ad libidum (Mouse Breeding, High Energy, ssniff, cat. no. V1185-300) at the animal facility at the Max Planck Institute of Immunobiology and Epigenetics Freiburg. Age- and sex-matched (8–25 weeks) male and female animals were used for experiments using knockout strains. Cre-expressing littermate control animals served as controls when possible. A contribution of Cre expression to biological phenotypes was controlled for and has not been observed. Male (8–12 weeks) C57BL/6J mice were used for bone marrow isolations for all other experiments. Peritoneal macrophages were isolated from aged (114–116 weeks) and young (8–15 weeks) female C57BL/6J mice. Aged mice were obtained through the shared animal programme for animal welfare at the Max Planck Institute of Immunobiology and Epigenetics.

### Antibodies and reagents

The following antibodies were used for immunofluorescence staining (IF), western blot (WB) or flow cytometry (FACS): anti-ABCD1 (WB 1:5,000; IF 1:500; Abcam, ab197013), anti-ACSL1 (IF 1:200, Proteintech, 13989-1-AP), anti-ATGL (WB 1:1,000; IF 1:200; Cell Signaling Technology, 2439), anti-β-actin-horseradish peroxidase (HRP) (WB 1:40,000; SantaCruz, sc-47778 HRP), anti-Calnexin (IF 1:250; Proteintech, 10427-2-AP), anti-Catalase (IF 1:300; FACS 1:100; R&D Systems, AF3398), anti-CD16/CD23 (FACS 1:1,000; Thermo Fisher, 14-0161-82), anti-CD107a (IF 1:200; BioLegend, 121601), anti-CD107a Alexa488-conjugated (FACS 1:200; BioLegend, 121607), anti-COX2 (IF 1:400; WB 1:1,000; Cell Signaling Technology, 12282), anti-cPLA2 (IF 1:250; WB 1:1,000; Cell Signaling Technology, 5249), anti-DRP1 (WB 1:1,000, IF 1:100; Cell Signaling Technology, 8570), anti-phospho-DRP1 (S616) (WB 1:1,000; Cell Signaling Technology, 3455), anti-phospho-DRP1 (S637) (WB 1:1,000; Cell Signaling Technology, 4867), anti-FAM73B (WB 1:1,000; Abcam, ab122713), anti-GM130 (IF 1:300; FACS 1:500; BD Bioscience, 610823), anti-GNPAT (WB 1:1,000; Proteintech, 14931-1-AP), anti-HSD17B4 (WB 1:1,000; Novus Biologicals, NBP1-85296), anti-HSP60 (IF 1:1,000; FACS 1:500; Cell Signaling Technology, 12165), anti-HSP60 (IF 1:1,000, antibodies.com, A85438), anti-MFN2 (WB 1:1,000; Abcam, ab124773), anti-mPGES1 (IF 1:500; WB 1:1,000; Abcam, ab180589), anti-OPA1 (WB 1:1,000; Thermo Fisher, MA5-16149), anti-PEX5 (WB 1:5,000; Novus Biologicals, NBP1-87185), anti-RMDN3 (WB 1:200; Thermo Fisher, PA5-117028), anti-rabbit HRP (WB 1:8,000; ThermoFisher, 31460), anti-goat HRP (WB 1:10,000; ThermoFisher, 31402), anti-mouse HRP (WB 1:10,000; ThermoFisher, 61-6520), anti-rabbit biotin-conjugated (IF 1:500; Thermo Fisher, A16039), anti-rabbit Cy3 (IF 1:1,000; Jackson Immuno Research Laboratories, 111-165-144), anti-rabbit Alexa Fluor 568 (IF 1:500; Thermo Fisher, A10042), anti-rabbit Alexa Fluor 647 (IF 1:500, FACS 1:500; Thermo Fisher, A31573), anti-mouse DyLight 405 (IF 1:300; Jackson Immuno Research Laboratories, 715-475-151), anti-mouse Alexa Fluor 568 (IF 1:500, FACS 1:500; Thermo Fisher, A10037), anti-goat DyLight 405 (IF 1:300; Jackson Immuno Research Laboratories, 705-475-147), anti-goat Alexa 405 Plus (IF 1:500; Thermo Fisher, A48258), anti-goat Alexa Fluor 568 (IF 1:500; Thermo Fisher, A11057) anti-goat Alexa Fluor 647 (IF 1:500, FACS 1:500; Thermo Fisher, A32849), anti-rat DyLight 550 (IF 1:600; Thermo Fisher, SA5-10027) and anti-chicken Alexa Fluor 647 (IF 1:500, Jackson Immuno Research Laboratories, 703-605-155).

The following dyes and chemicals were used: BODIPY ^493/503^ (500 ng ml^−1^ fixed, 200 ng ml^−^^1^ live imaging, D3922), BODIPY ^558/568^ C_12_ (1 µM 24 h pulse, 5 µM acute pulse, D3835), CellROX Deep Red (C10422), Fixable Viability Dye eFluor 450 (65086314), Fixable Viability Dye eFluor 780 (65086514), MitoSOX red (5 µM, M36008), MitoTracker Green FM (100 nM, M7514), Streptavidin Alexa Fluor 514 conjugate (IF 1:1,500; S32353) and tetramethylrhodamine methyl ester perchlorate (T668) from Thermo Fisher, LipidSpot 610 from Biotium (70069), MitoTracker Deep Red FM from Cell Signaling Technology (50 nM, 8778P), IFNγ (50 ng ml^−1^; PeproTech, AF-315-05), LPS (20 or 100 ng ml^−1^; InvivoGen, tlrl-pb5lps), macrophage colony stimulating factor (20 ng ml^−1^; PeproTech, 315-02), *S. aureus* BioParticles (3 × 10^6^ particles ml^−1^; Thermo Fisher, S2859) and ATGL (20 µM; 40 µM only Extended Data Fig. 6e,f; SML1075), Etomoxir (25 µM; E1905), Nocodazole (5 µM; M1404), PF-06424439 (PZ0233, 10 µM) and T863 (75 µM, SML0539) (Sigma-Aldrich).

### BMDM differentiation and activation

Bone marrow was isolated from pelvis, femur and tibia of mice and differentiated for 6 days in BMDM medium (RPMI 1640, 10% FCS, 100 U ml^−1^ penicillin and 0.1 mg ml^−1^ streptomycin) containing 20 ng ml^−1^ macrophage colony stimulating factor at 37 °C and 5% CO_2_. BMDMs were activated with 20 ng ml^−1^ LPS and 50 ng ml^−1^ IFNγ. For heat-killed *S. aureus* treatment, BMDMs were activated with 1 × 10^6^ bacterial particles spun on cells by centrifugation for 30 s at 300*g* and incubated for 30 min and collected after 24 h. For nocodazole treatment, BMDMs were activated for 23 h in BMDM activation medium. The cells were transferred to ice for 2 min, the medium was exchanged for BMDM medium containing 5 µM nocodazole and the cells were incubated for 1 h at 37 °C and 5% CO_2_.

### Isolation of peritoneal macrophages

The peritoneal cells were collected by lavage and cell suspensions cultured for 2.5 h in RPMI supplemented with macrophage colony stimulating factor (20 ng ml^−1^) allowing macrophages to adhere. The non-adherent cells were washed off five times with cold PBS. The adherent cells were collected using 20 mM EDTA and plated in BMDM medium. Peritoneal macrophages were activated for experiments with BMDM activation medium.

### Plasmids

New plasmids were generated using the CloneAmp HiFi PCR Premix and In-Fusion HD Cloning Kit (Takara) or the NEBuilder HiFi DNA Assembly kit (New England Biolabs) according to the respective manufacturer’s instructions. The following original sequences and target vectors were used: ERmoxGFP (Addgene no. 68072) and pBMN backbone (Addgene no. 1734), ERmoxVenus, mOrange2Peroxisomes2 (Addgene no. 54596), *Abcd1* (Dharmacon no. MMM1013-202798649), and MigR1-IRES-GFP backbone (Addgene no. 27490), *Miga2* (Dharmacon no. MMM1013-202770174) and MigR1-IRES-BFP plasmid, mApple-M1-Spastin (Addgene no. 134461) and pBMN plasmid, human *Mfn2* (Addgene no. 121997) and *Rmdn3* (Addgene no. 170536) and MigR1-IRES-BFP backbone. The MigR1-Drp1K38A-IRES-GFP and MigR1-Drp1K38A-IRES-BFP plasmids were generated inserting the Drp1K38A sequence into the MigR1-IRES-GFP and MigR1-IRES-BFP backbones, respectively. The Drp1^CA^ mutant (Drp1-S579E-S600A) was generated by mutagenesis of the MigR1-Drp1K38A-IRES-BFP plasmid. The primers used to amplify or delete the respective DNA sequences are listed in Supplementary Table 1.

SMARTvector lentiviral shRNA plasmids targeting *Abcd1* (no. V3SM11241-233039389), *Gnpat* (no. V3SM11241-234930851), *Mfn2* (no. V3SM11241-235275969), *Mfp2* (no. V3SM11241-235172019) or *Rmdn3* (no. V3SM11241-231389259) and a SMARTvector non-targeting control (no. VSC11715) were obtained from Dharmacon Reagents.

### Mitochondrial DNA quantification

Mitochdondrial DNA was isolated using the PureLink genomic DNA Mini Kit (Invitrogen, K1820-01) according to the manufacturer’s instructions. Mitochondrial and nuclear DNA content quantified was with primers for *Mtco1* and *Ndufv1*, respectively (Supplementary Table 2). The samples were measured in the 7500 Fast Real-Time PCR System (Applied Bioscience) and analysed via StepOne Software (AB, v.2.0). The ratio of mitochdondrial DNA to nuclear DNA was calculated (2^ct nDNA^/2^ct mtDNA^).

### Immunofluorescence

Macrophages were seeded on fibronectin-coated 12-mm glass coverslips and treated as desired. The cells were fixed with 4% paraformaldehyde 15 min at room temperature and permeabilized with 0.2% Triton X-100 in PBS. If biotinylated antibodies were used, endogenous biotin was blocked by using the Endogenous Biotin-Blocking Kit (Invitrogen, E21390) according to the manufacturer’s instructions. Subsequently, the coverslips were incubated in blocking solution (5% foetal bovine serum, 0.1% Tween-20 in PBS) for 1 h at room temperature. Primary and secondary antibody staining were performed in blocking buffer for 16 h at 4 °C and 1 h at room temperature, respectively. Streptavidin staining was performed in PBS for 1 h at room temperature subsequent to secondary antibody staining. To visualize lipid droplets, samples were incubated for 10 min at room temperature with 500 ng ml^−1^ Bodipy^493/502^ or LipidToxRed (1:1,000). The samples were mounted in Fluoromount-G (Thermo Fisher, 00-4959-52) or ProLong Glass Antifade Mountant (Thermo Fischer, P36982) for structured illumination microscopy (SIM) applications. Staining panels for each figure are listed in Supplementary Information (Supplementary Table 3).

### Confocal microscopy, structured illumination microscopy and image processing

Spectral imaging was conducted on a LSM780 Zeiss confocal microscope using a 32-channel QUASAR detector (Carl Zeiss) in lambda mode with 8.9 nm bins from 410 to 695 nm, a 63×/1.4 NA objective lens and ZEN 2012 SP5 black software (Carl Zeiss, v14.0.26.201). The fluorophores were simultaneously excited using 405, 488, 561 and 633 nm lasers and 405 and 488/561/633 nm main beam splitters. OrgaPlex primary–secondary antibody combinations were chosen to be devoid of cross-reactivity to other antibody pairs and to show highly specific organelle with minimal background staining. Antibody pairs and dyes were carefully curated to ensure proper visualization of all six organelles without overexposing pixels in one or more emission band widths. Spectral unmixing was performed using single spectra of fluorescent reporters defined by single staining and the ZEN 2012 SP5 black software. Unmixed images were deconvolved using Huygens Professional software (Scientific Volume Imaging, v22.10.0p1) and theoretical point-spread functions. All other confocal images were acquired with an inverted LSM780 or LSM880 Zeiss confocal microscope equipped with a 63×/1.4 NA or 40×/1.4 NA objective lens and ZEN 2012 SP5 black or ZEN 2.3 SP1 FP3 black (Carl Zeiss, v14.0.27.201), respectively. To quantify mitochondrial morphology, images were oversampled (*x*/*y*/*z* 0.08/0.08/0.2 µm) for optimal deconvolution using Huygens Professional software with theoretical point-spread functions. Structured illumination microscopy was conducted using an Elyra 7 super-resolution microscope (Carl Zeiss) equipped with a 63×/1.4 NA objective lens. The images were acquired as small LD-encompassing *Z*-stacks using three grating rotations. Super-resolution images were generated using the structured illumination tool of the ZEN software (Carl Zeiss, ZEN black v16.0.15.306). Live cell imaging experiments were conducted at 37 °C and 5% CO_2_ using the LSM780 or LSM880 confocal microscopes and a stage-top incubator (TokaiHit). Spectral live cell imaging (ERmoxVenus, mOrange2-Peroxisomes2, MitotrackerDeepRed FM, 200 ng ml^−1^ BODIPY^493/503^) was performed at 37 °C. Individual focal planes were acquired in lambda mode (8.9 nm bins) in intervals of 10 s. For acute fatty acid pulse-chase experiments, the images were acquired in 5–6 s intervals. When required for imaging analysis, images were spatially deconvolved using Huygens Professional as described above. The depicted images were prepared using Imaris (Bitplane, v.9.7.2), and brightness and contrast were adjusted. A median filter was applied. The images depicting maximum intensity projections of ER stainings were gamma-adjusted to better visualize peripheral ER structures. Background subtraction was applied for images of Figs. 1b,c and 2a and Extended Data Figs. 3d and 6g.

### Image analysis

Organelle positioning and proximity were analysed using the 3D surface objects tool of Imaris (Bitplane, v.9.7.2). Regions of interest were defined for the centre and periphery of each cell. The threshold values were manually adjusted to optimally segment each organelle. The split-touching-objects function was applied to optimally analyse organelle positioning and HPS formation for bigger organelle structures. Although this approach overestimates surface numbers for organelles with big, continuous surfaces (for example, the ER and mitochondria), it generated a better estimation of cellular organelle distribution and HPS formation compared with segmenting large surface objects. Especially for mitochondria, this also allowed the analysis of organelle–organelle contacts irrespective of tubulated or fragmented organelle morphologies. The following smoothing and seed-point diameters were used: ER 0.02 and 0.2, Golgi 0.03 and 0.3, LD 0.04 and 0.4–0.5 (depending on LD size), lysosome 0.02 and 0.2, mitochondria 0.03 and 0.3 and peroxisome 0.03 and 0.3, respectively. Imaris statistic data were further processed using custom code generated in R studio (v1.3.1093).

For distance measurements, the nucleus centre was manually determined and surface volumes determined over a 3D coordinate system. The surface volume of each organelle was summed up in bins of 0.25 µm (from nucleus centre) and averaged across 15 cells per condition. Organelle proximity was determined with Imaris surface nearest neighbour analysis, surface edge to surface edge tool. Distances ≤0 nm were defined as HPS. For pairwise interaction analysis, HPS for the bait organelle with the higher surface number were depicted as the reciprocal analysis generally showed the same trend. The network graph was generated using ggraph (2.0.6) and tidygraph (V1.2.2) packages with R Studio (V 2022.07.1.554). LD–organelle clusters are LD-surface objects that form HPS with multiple organelle classes at the same time. LD–organelle clusters with an average frequency <0.8 were considered artefacts or too low abundant and excluded from analysis.

Organelle mass (total volume per cell), mitochondrial morphology and sphericity, overlapped volume between organelles and total LD surface area per cell were quantified using the Imaris surface tool. Co-localization of LDs with organelles and 3D representations of co-localized pixels were generated with the Imaris co-localization tool. Co-localization of DRP1 with mitochondria (HSP60) was quantified using the Pearson’s Coefficient ImageJ Jacob co-localization software tool (https://imagej.net/ij/plugins/track/jacop.html). Proximity sites of mitochondria and peroxisomes to LDs in living cells were analysed using Fiji ImageJ (V2.1.0). Individual mitochondria or peroxisomes were manually tracked over time and HPS to LDs were manually counted. The organelles that did not contact LDs were excluded. Contact plots (heat maps) indicate proximity sites of tracked organelles with LDs over time. Peroxisomal RedC12 signal was quantified in two-dimensional surfaces based on the Emerald-Peroxisomes2 signal (average MFI per cell) using the Imaris surface tool.

### TG lipidomics

TGs were measured using liquid chromatography (LC) quadrupole time-of-flight (QTOF) mass spectrometry (MS).

Cells were lysed in ice-cold methanol (750 µl) and methyl-*tert*-butyl ether (2.5 ml) and samples incubated at 4 °C for 1 h under shaking. The phases separated by the addition of H_2_O (625 µl) and centrifugation at 1,000*g* for 10 min. Organic phases were dried in a Genevac EZ-2 (SP Scientific). The samples were resuspended in 40 µl of a 2:1:1 2-propanol:acetonitrile:H_2_O mixture and measured using an Agilent 1290 infinity II UHPLC system coupled to a Bruker impact II QTOF–MS as previously described^61^. In brief, scan range was from 50 to 1,600 Da. Mass calibration was performed at the beginning of each run. LC separation occurred on a Zorbax Eclipse plus C18 column (100 mm × 2 mm, 1.8 µm particles) using a solvent gradient of 70% buffer A (10 mM ammonium formiate in 60:40 acetonitrile:water) to 97% buffer B (10 mM ammonium formiate in 90:10 2-propanol:acetonitrile). The flow rate was 400 µl min^−1^, autosampler temperature was 5 °C and injection volume was 2 µl. The lipid profiles were analysed using MetaboScape Software (version 2023, Bruker), and the lipid species were annotated using the spectral library and lipid species functions. Levels of TGs containing the fatty acid C20:4 were quantified as arbitrary units.

### Metabolic tracing

Metabolic tracing was conducted using gas chromatography (GC) coupled to MS. For glucose tracing, BMDMs were incubated for 24 h in glucose-free BMDM medium supplemented with 11 mM ^13^C-glucose. For palmitate tracing, BMDMs were incubated with 20 µM BSA reagent-conjugated ^13^C-palmitic acid for the last 18 h of activation. To extract metabolites, BMDMs were washed once with ice-cold 0.9% NaCl in MilliQ-H_2_O, shock frozen in an ethanol–dry ice bath and collected with a cell lifter in ice-cold 80% methanol. The cell debris was removed by centrifugation for 5 min at 20,000*g* and 4 °C. Methanol supernatants were collected and dried in a Genevac EZ-2 (SP Scientific). The metabolites were resuspended in 10 µl D27/methoxyamine mix (10 mg ml^−1^ methoxyamine hydrochloride, 0.2 µg ml^−1^ myristic-d_27_ acid in pyridine) for 1 h at 30 °C, and the mix were derivatized with *N*-(*tert*-butyldimethylsilyl)-*N*-methyl-trifluoroacetamid, with 1% *tert*-butyldimethyl-chlorosilane (375934 Sigma-Aldrich) for 60 min at 80 °C. Isotopomer distributions were measured using a DB5-MS GC column (30 m × 0.25 mm) in a 7890 GC system (Agilent Technologies) combined with a 5977 MS system (Agilent Technologies). Metabolic tracing of ^13^C-palmitate into lipids was conducted using LC–QTOF–MS. BMDMs were pre-treated for 4 h in BMDM activation medium and subsequently incubated in BMDM medium containing 20 µM BSA-conjugated ^13^C-palmitic acid for 20 h. The samples were collected and processed as described in the lipidomics section. Data pre-processing for metabolic tracing experiments was performed in R as described previously^62,63^. Data from tracing experiments are presented as ^13^C-labelled metabolite fractions of respective total metabolite level.

### PGE_2_ measurements

PGE_2_ levels in BMDM supernatants were determined using enzyme-linked immunosorbent assays (Abcam, ab133021, discontinued; R&D Systems, SKGE004B) according to the respective manufacturer’s instructions. Absorbance was detected using a SpextraMax250 microplate reader (Molecular Devices). Alternatively, PGE_2_ levels were quantified using targeted LC–MS. The macrophage supernatant was incubated with acetonitrile (1:2 v/v) overnight at 4 °C. The precipitated proteins were pelleted by centrifugation at 3,000*g* for 10 min, and the supernatant was transferred to H_2_O (1:2 v/v). Prostaglandin LC–MS was carried out using an Agilent 1290 Infinity II UHPLC in-line with an Agilent 6495 triple-quadrupole–MS operating in MRM mode. The selected prostaglandins were used as pure standards and transferred for the detection of their respective isomers. LC separation was on a Waters Acquity HSS PFP column (100 mm × 2.1 mm, 1.7 µm particles) using a solvent gradient of 75% buffer A (0.1% v/v formic acid in water) to 90% buffer B (acetonitrile). The flow rate was 400 µl min^−1^. The autosampler temperature was 6 °C. Data processing was performed using Agilent MassHunter Software (version 8).

### RNA-seq

RNA isolation was performed using the RNeasy MinElute Cleanup Kit according to the manufacturer’s instructions. Complementary DNA libraries were prepared by the Deep Sequencing facility at the Max Planck Institute of Immunobiology and Epigenetics using the TrueSeq stranded mRNA protocol (Illumina) and sequenced on a HiSeq 3000 (Illumina) platform to a depth of 16 million reads per sample. Initial RNA-seq analysis was performed with snakePipes (version 2.7.2)^64^ with the mRNA-seq module. Raw fastq files were trimmed for adaptors using cutadapt (version 4.1) and aligned to the mm10 reference genome with STAR (version 2.7.10b). The gene counts were generated using featureCounts (version 2.0.1)^65^ using gene annotations from gencode version M19^66^. Sequencing quality control was performed using deeptools (version 3.5.1)^67^. The gene expression analysis was performed using DESeq2 (version 1.34.0)^68^, with a design of the form ~batch + timepoint. Differentially expressed genes over different timepoints were identified using the likelihood ratio test in DESeq2, using ~batch as the reduced model. The results with a false discovery rate ≤0.05 were considered significant. For visualization purposes, normalized counts (generated with the vst function implemented in DESeq2) were corrected for batch effects using the removeBatchEffect function implemented in limma (version 3.50.3)^69^.

### Production of viral particles and BMDM transduction

Retroviral particles were produced transfecting PlatinumE packacking cells with the respective retrovial expression vectors using Lipofectamine3000 (Thermo Fischer) according the manufacturer’s instructions. For shRNA-mediated protein knock-down, lentiviral particles were produced by the transfection of 293T HEK cells with a combination of the lentiviral packaging vectors psPAX2 and pCAG-eco and the respective shRNA-encoding plasmid using Lipofectamine3000. Retro- and lentivirus-containing supernatant was collected every 24 h for 4 days. Bone marrow cultures were transduced on day 2 of BMDM differentiation by the addition of viral particles (1:3 dilution in BMDM medium). After 18 h, virus-containing medium was exchanged for BMDM medium, and the cells were cultured until fully differentiated. If required, virus-targeted BMDMs were sorted using the BD FACSAria III cell sorter FACSDiva (BD, v.8.0.1).

### Western blotting

BMDMs were lysed for 15 min on ice with lysis buffer (50 mM Tris, 150 mM NaCl, 0.1% Triton X-100, pH 7.4) supplemented with 1× Halt Protease Inhibitor Cocktail and 1× Phosphatase Inhibitor Cocktail (Thermo Fischer) and sheared with a 26G insulin syringe. A total of 25 µg of total protein were separated by SDS–polyacrylamide gel electrophoresis on 10% or 4–20% gradient polyacrylamide gels (Bio-Rad, 456-1094). Semi-dry protein transfer to a polyvinyl difluoride-membrane (Merck Millipore) was performed for 45–75 min at 10 V. Membranes were blocked for 1 h in 5% milk in Tris-buffered saline with 0.1% Tween (TBST) at room temperature. Primary antibody staining was performed overnight at 4 °C in TBST containing 2–5% BSA. Incubation with secondary antibodies was performed for 1 h at room temperature in 5% milk in TBST. The protein signals were detected using Amersham ECL Prime Western Blotting Detection Reagent and the ChemiDoc Touch Gel Imaging System (Bio-Rad). The images were prepared with the Image Lab v.5.2 TM Touch Software (Bio-Rad, v.1.0.0.15).

### Flow cytometry analysis

BMDMs were stained with: 100 nM MitotrackerGreen, 5 µM MitoSOX, 50 nM TMRM or 0.5 µM CellROX and and DAPI (0.5 µg ml^−1^). The cells were subsequently washed three times with prewarmed phenol-fee BMDM medium. To validate the expression of maker proteins used for spectral imaging, activated and control BMDMs were collected with 20 mM EDTA, incubated for 10 min on ice with Live Dead Fixable Viability eFluor780 or eFluor450 (1:1,000) in PBS and fixed using the Foxp3 transcription factor staining buffer set (eBioscience, 00552300). The samples were then incubated in wash buffer containing 5% FCS and CD16/32 blocking antibodies (1:1,000, BD Biosciences) for 1 h at room temperature and subsequently stained with primary antibodies in wash buffer for 1 h at room temperature. Secondary antibody staining was performed in wash buffer for 1 h at room temperature. All samples were measured on the BD LSR Fortessa cell analyser (BD Biosciences) data were analysed in FlowJo (BD Biosciences, v.10.7.1).

### Statistics and reproducibility

For all experiments, no statistical method was used to pre-determine sample size. Sample sizes are directly indicated in the figure legends. The imaging data were excluded from analysis when poor staining quality did not allow image acquisition, spectral unmixing or analysis. PGE_2_ measurements of BMDMs derived from knockout animals were excluded when a knockout could not be confirmed by western blotting or immunofluorescence analysis. C57BL/6 mice were randomly assigned from the breeding facility by the facility staff without knowledge of the experimental set-up for which the mice were intended. No other randomization has been performed. For image acquisition, FACS, metabolomic and TG analysis with BMDMs derived from knockout animals, investigators were blinded to allocation during experiments and outcome assessment. Mouse ID numbers were used as identifiers. No blinding occurred for all other experiments. Statistical analysis was performed using GraphPad Prism v.9.5.1. Statistical tests used are indicated in the figure legends and in Supplementary Information (Supplementary Table 4). The *P* values < 0.05 were considered statistically significant. Statistical differences are indicated as asterix (*P ≤ 0.05, **P ≤ 0.01, ***P ≤ 0.001, ****P ≤ 0.0001) or non-significant. Numeric *P* values are listed in in Supplementary Table 4. Heat maps were generated with Morpheus (Broad Institute). For two paragraphs, ChatGPT was used to improve English language. The schematics were generated with BioRender and Adobe Illustrator (Adobe, v25.0.1) and figures with Adobe Illustrator.

### Materials availability

No new, unique reagents, plasmids or mice were generated in this study.

### Reporting summary

Further information on research design is available in the Nature Portfolio Reporting Summary linked to this article.

## Online content

Any methods, additional references, Nature Portfolio reporting summaries, source data, extended data, supplementary information, acknowledgements, peer review information; details of author contributions and competing interests; and statements of data and code availability are available at 10.1038/s41556-024-01457-0.

### Supplementary information

Supplementary Information Supplementary FACS gating strategy and the corresponding figure legend.

Reporting Summary

Supplementary Data 1 Extended source data for TG lipidomics.

Supplementary Table 1 Table 1: used cloning primers. Table 2: used mtDNA primers. Table 3: organelle staining table. Table 4: statistics file including numerical *P* values for all relevant experiments.

### Source data

Source Data Fig. 1 Statistical source data.

Source Data Fig. 2 Statistical source data.

Source Data Fig. 3 Statistical source data.

Source Data Fig. 4 Statistical source data.

Source Data Fig. 5 Statistical source data.

Source Data Fig. 6 Statistical source data.

Source Data Extended Data Fig. 1 and Table 1 Statistical source data.

Source Data Extended Data Fig. 2 and Table 2 Statistical source data.

Source Data Extended Data Fig. 2 and Table 2 Unprocessed WBs.

Source Data Extended Data Fig. 3 and Table 3 Statistical source data.

Source Data Extended Data Fig. 4 and Table 4 Statistical source data.

Source Data Extended Data Fig. 5 and Table 5 Statistical source data.

Source Data Extended Data Fig. 6 and Table 6 Statistical source data.

Source Data Extended Data Fig. 7 and Table 7 Statistical source data.

Source Data Extended Data Fig. 7 and Table 7 Unprocessed WBs.

Source Data Extended Data Fig. 8 and Table 8 Statistical source data.

Source Data Extended Data Fig. 8 and Table 8 Unprocessed WBs.

Source Data Extended Data Fig. 9 and Table 9 Statistical source data.

Source Data Extended Data Fig. 9 and Table 9 Unprocessed WBs.

Source Data Extended Data Fig. 10 and Table 10 Statistical source data.

Source Data Extended Data Fig. 10 and Table 10 Unprocessed WBs.

## Data Availability

RNA-sequencing data generated in this study have been deposited in the Gene Expression Omnibus (GEO) database and are available under the accession code GSE235484. The TG lipidomics data generated in the study can be found in Supplementary Data. Source data are provided with this paper. All other data supporting the findings of this study are available from the corresponding author on reasonable request.
