# Supplementary material for: Functional multi-organelle units control inflammatory lipid metabolism of macrophages
Source: Nat Cell Biol. 2024 Jul 5;26(8):1261–73. doi: 10.1038/s41556-024-01457-0 (PMC11321999; doi:10.1038/s41556-024-01457-0)
Supplement: Supplementary file 1 — Supplementary FACS gating strategy and the corresponding figure legend. [file 41556_2024_1457_MOESM1_ESM.pdf]

nature cell biology

Supplementary information

https://doi.org/10.1038/s41556-024-01457-0

Functional multi-organelle units control inflammatory lipid metabolism of macrophages

In the format provided by the authors and unedited

Supplementary Information Figure

a gating strategy for intracellular staining of organelle markers used for OrgaPlexing

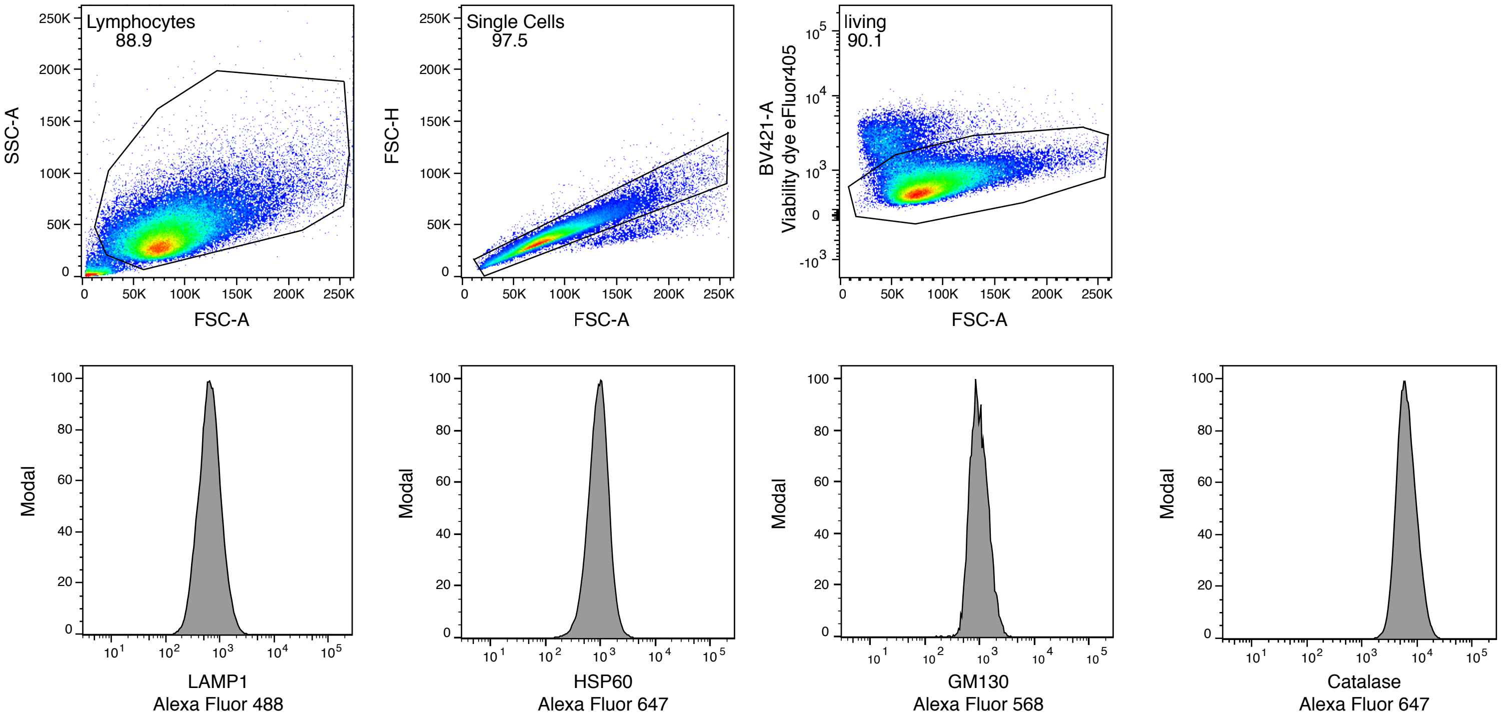

b gating strategy for living cells staindled with CellROX, MitoSOX, MitotrackerGreen and TMRM

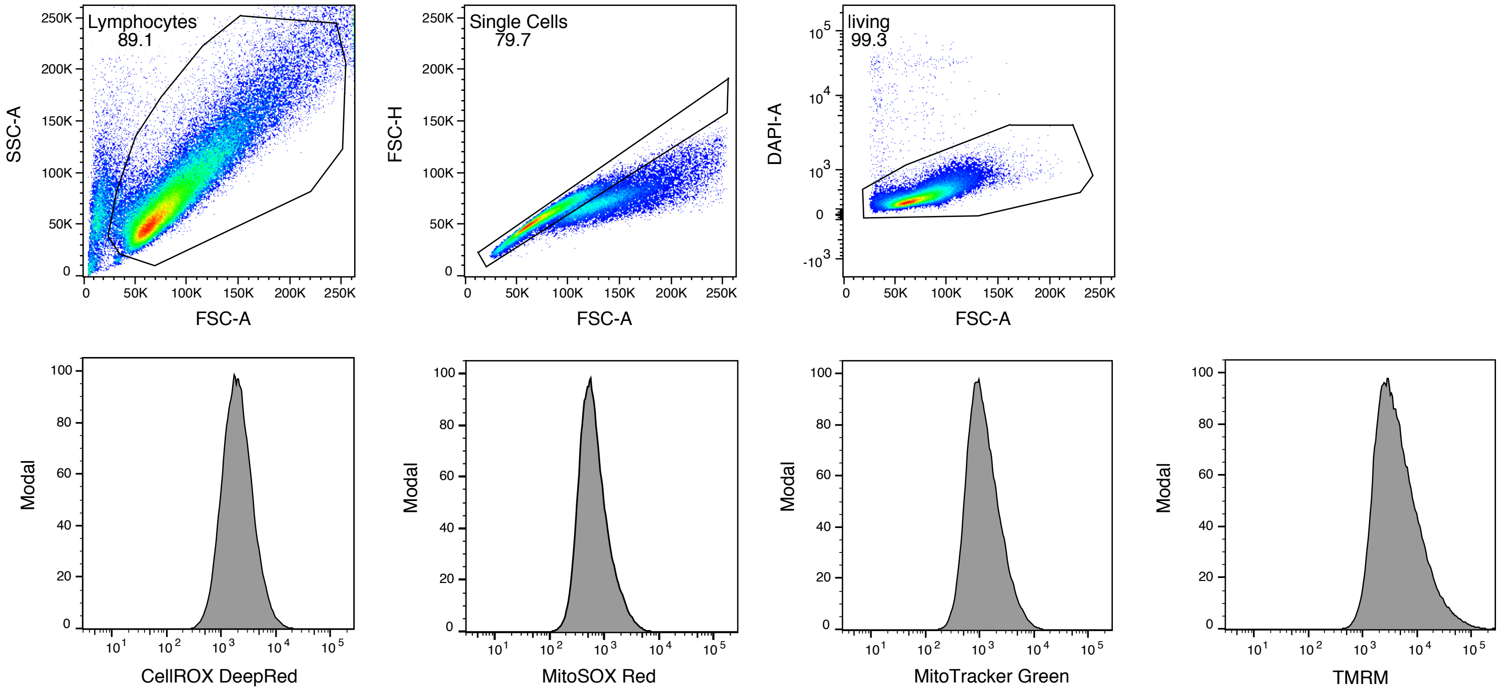

Supplemental Information Fig. 1: Gating strategy for flow cytometry analysis. a Gating strategy for organelle markers used for OrgaPlexing. BMDMs were gated for single cells, that were negative for Viability Dye eFluor405 (top). Representative histograms of organelle marker stainings for LAMP1, HSP60, GM130 and Catalase (bottom). **b** Gating strategy for BMDMs stained with intracellular dyes. BMDMs were gated for single cells, that were negative for DAPI (top). Representative histograms of CellROX, MitoSOX, MitoTracker and TMRM stainings (bottom).
