# Supplementary material for: Functional multi-organelle units control inflammatory lipid metabolism of macrophages
Source: Nat Cell Biol. 2024 Jul 5;26(8):1261–73. doi: 10.1038/s41556-024-01457-0 (PMC11321999; doi:10.1038/s41556-024-01457-0)
Supplement: Supplementary file 2 — Reporting Summary [file 41556_2024_1457_MOESM2_ESM.pdf]

nature portfolio

Corresponding author(s): Angelika S. Rambold

Last updated by author(s): 22nd of May 2024

Reporting Summary

Nature Portfolio wishes to improve the reproducibility of the work that we publish. This form provides structure for consistency and transparency in reporting. For further information on Nature Portfolio policies, see our Editorial Policies and the Editorial Policy Checklist.

Statistics

For all statistical analyses, confirm that the following items are present in the figure legend, table legend, main text, or Methods section.

n/a	Confirmed	
☐	☒	The exact sample size (*n*) for each experimental group/condition, given as a discrete number and unit of measurement
☐	☒	A statement on whether measurements were taken from distinct samples or whether the same sample was measured repeatedly
☐	☒	The statistical test(s) used AND whether they are one- or two-sided<br>*Only common tests should be described solely by name; describe more complex techniques in the Methods section.*
☒	☐	A description of all covariates tested
☒	☐	A description of any assumptions or corrections, such as tests of normality and adjustment for multiple comparisons
☐	☒	A full description of the statistical parameters including central tendency (e.g. means) or other basic estimates (e.g. regression coefficient) AND variation (e.g. standard deviation) or associated estimates of uncertainty (e.g. confidence intervals)
☐	☒	For null hypothesis testing, the test statistic (e.g. *F*, *t*, *r*) with confidence intervals, effect sizes, degrees of freedom and *P* value noted<br>*Give P values as exact values whenever suitable.*
☒	☐	For Bayesian analysis, information on the choice of priors and Markov chain Monte Carlo settings
☒	☐	For hierarchical and complex designs, identification of the appropriate level for tests and full reporting of outcomes
☐	☒	Estimates of effect sizes (e.g. Cohen's *d*, Pearson's *r*), indicating how they were calculated

Our web collection on statistics for biologists contains articles on many of the points above.

Software and code

Policy information about availability of computer code

Data collection	Imaging data collection: ZEN 2012 SP5 black software (Carl Zeiss, v14.0.26.201) , ZEN 2.3 SP1 FP3 black (Carl Zeiss, v14.0.27.201), Zen black (Carl Zeiss v16.0.15.306)<br>FACS data collection: FACS Diva (BD Biosciences, v8.0.1)<br>Western Blot data collection: Image Lab 5.2 TM Touch Software (Bio-Rad, v1.0.0.15)<br>Real-time PCR data collection: StepOne Software (Applied Biosciences, v2.0)<br>RNA Seq data collection: HiSeq 3000 (Illumina)<br>PGE2 ELISA data collection: SoftMax Pro Software (Molecular Devices, v5.4.1)
Data analysis	Statistical data analysis and gegeration of graphs: Graphpad Prism (v9.5.1)<br>Imaging data analysis: Imaris (Bitplane, v9.7.2), Huygens Professional (Scientific Volume Imaging, v22.10.0p1), Fiji ImageJ (v2.1.0/1.53g), ImageJ Jacob colocalization software tool (https://imagej.net/ij/plugins/track/jacop.html), R Studio (v1.3.1093 and V2022.07.1.554), ggraph (2.0.6), tidygraph (V1.2.2), Microsoft Excel for Mac (v16.66.1)<br>FACS data analysis: FlowJo (BD Biosciences, v10.7.1)<br>Real-time PCR analysis: Microsoft Excel for Mac (v16.66.1)<br>Western Blot analysis:Fiji ImageJ (v2.1.0/1.53g)<br>RNA Seq analysis: snakePipes (version 2.7.2), cutadapt (version 4.1), STAR (version 2.7.10b), featureCounts (version 2.0.1), deeptools (version 3.5.1) , DESeq2 (version 1.34.0), limma (version 3.50.3)<br>PGE2 ELISA analysis: SoftMax Pro Software (Molecular Devices, v5.4.1), Microsoft Excel for Mac (v16.66.1)<br>Metabolomics: Bruker MetaboScape Software (Version 4, 2023)

For manuscripts utilizing custom algorithms or software that are central to the research but not yet described in published literature, software must be made available to editors and reviewers. We strongly encourage code deposition in a community repository (e.g. GitHub). See the Nature Portfolio guidelines for submitting code & software for further information.

Data

Policy information about availability of data

All manuscripts must include a data availability statement. This statement should provide the following information, where applicable:

- Accession codes, unique identifiers, or web links for publicly available datasets
- A description of any restrictions on data availability
- For clinical datasets or third party data, please ensure that the statement adheres to our policy

RNA-seq data generated in this study have been deposited in the GEO database and are available under the accession code GSE235484. The triglyceride lipidomics data generated in the study can be found in the Supplementary data files. Source data have been provided in Source Data. All other data supporting the findings of this study are available from the corresponding author on reasonable request.

Research involving human participants, their data, or biological material

Policy information about studies with human participants or human data. See also policy information about sex, gender (identity/presentation), and sexual orientation and race, ethnicity and racism.

Reporting on sex and gender	N/A
Reporting on race, ethnicity, or other socially relevant groupings	N/A
Population characteristics	N/A
Recruitment	N/A
Ethics oversight	N/A

Note that full information on the approval of the study protocol must also be provided in the manuscript.

Field-specific reporting

Please select the one below that is the best fit for your research. If you are not sure, read the appropriate sections before making your selection.

☒ Life sciences ☐ Behavioural & social sciences ☐ Ecological, evolutionary & environmental sciences

For a reference copy of the document with all sections, see nature.com/documents/nr-reporting-summary-flat.pdf

Life sciences study design

All studies must disclose on these points even when the disclosure is negative.

Sample size	For all experiments no statistical method was used to predetermine sample size. Sample sizes were estimated based on experience made by previous experiments (PMID:.PMID: 35864246, PMID: 32796836). For imaging, metabolomics, FACS, RT-PCR and WB experiments we used 3-4 independent experiments. This number of independent experiments is a standard sample size to accurately detect differences in cell biology experiments. Confocal images and Western Blots are shown as representative images.
Data exclusions	For imaging analysis: Cells were excluded when poor staining quality did not allow image acquisition, spectral unmixing or analysis.<br>For ELISA: Data sets of KO BMDMs were excluded when a KO could not be confirmed by Western Blot or immune fluorescence.
Replication	Reproducibility of the experimental findings was verified using biological replicates and independent experiments. The respective numbers are indicated in the figure legends.
Randomization	For BMDM cultures, C57BL/6 mice were randomly assigned from the breeding facility by the facility staff without knowledge of the experimental set-up for which the mice were intended.
Blinding	Experimentalists were blinded regarding the BMDM genotype. Mouse ID numbers were used as identifiers. Image acquisition and FACS analysis and metabolic measurements and analysis were performed blinded.

Reporting for specific materials, systems and methods

We require information from authors about some types of materials, experimental systems and methods used in many studies. Here, indicate whether each material, system or method listed is relevant to your study. If you are not sure if a list item applies to your research, read the appropriate section before selecting a response.

Materials & experimental systems

n/a	Involved in the study
☐	☒ Antibodies
☐	☒ Eukaryotic cell lines
☒	☐ Palaeontology and archaeology
☐	☒ Animals and other organisms
☒	☐ Clinical data
☒	☐ Dual use research of concern
☒	☐ Plants

Methods

n/a	Involved in the study
☒	☐ ChIP-seq
☐	☒ Flow cytometry
☒	☐ MRI-based neuroimaging

Antibodies

Antibodies used

Primary antibodies:
1. anti-ABCD1 (WB 1:5000; IF 1:500; Abcam, ab197013, Anti-ABCD1/ALD antibody [EPR15929]),
2. anti-ACSL1 (IF 1:200, Proteintech, 13989-1-AP, ACSL1 Polyclonal antibody),
3. anti-ATGL (WB 1:1000; IF 1:200; CST, 2439, ATGL (30A4) Rabbit mAb),
4. anti-beta-Actin-HRP (WB 1:40000; SantaCruz, sc-47778 HRP),
5. anti-Calnexin (IF 1:250; Proteintech, 10427-2-AP, Calnexin Polyclonal antibody),
6. anti-CD16/CD23 (FACS 1:1000; Thermo Fisher, 14-0161-82, CD16/CD32 Monoclonal Antibody (93), eBioscience™),
7. anti-Catalase (IF 1:300; FACS 1:100; R&D Systems, AF3398, Human/Mouse/Rat Catalase Antibody),
8. anti-CD107a (IF 1:200; BioLegend, 121601, Purified anti-mouse CD107a (LAMP-1) Antibody, 1D4B),
9. anti-CD107a Alexa488-conjugated (FACS 1:200; BioLegend, 121607, Alexa Fluor® 488 anti-mouse CD107a (LAMP-1) Antibody, 1D4B),
10. anti-COX2 (IF 1:400; WB 1:1000; CST, 12282, Cox2 (D5H5) XP Rabbit mAb),
11. anti-cPLA2 (IF 1:250; WB 1:1000; CST, 5249, cPLA2 (D49A7) Rabbit mAb),
12. anti-Drp1 (WB 1:1000; IF 1:100; CST, 8570, DRP1 (D6C7) Rabbit mAb),
13. anti-phospho-Drp1 (S616) (WB 1:1000; CST, 3455, Phospho-DRP1 (Ser616) Antibody),
14. anti-phospho-Drp1 (S637) (WB 1:1000; CST, 4867, Phospho-DRP1 (Ser637) Antibody),
15. anti-FAM73B (WB 1:1000; Abcam, ab122713, Anti-FAM73B antibody),
16. anti-GM130 (IF 1:300; FACS 1:500; BD Bioscience, 610823, Purified Mouse Anti-GM130, 35/GM130),
17. anti-GNPAT (WB 1:1000; Proteintech, 14931-1-AP, GNPAT Polyclonal antibody),
18. anti-HSD17B4 (WB 1:1000; Novus Biologicals, NBP1-85296, HSD17B4 Antibody),
19. anti-Hsp60 (IF 1:1000; FACS 1:500; CST, 12165, HSP60 (D6F1) XP Rabbit mAb),
20. anti-HSP60 (IF 1:1000, antibodies.com, A85438)
21. anti-MFN2 (WB 1:1000; Abcam, ab124773, Anti-Mitofusin 2 antibody [NIAR164]),
22. anti-MPGES1 (IF 1:500; WB 1:1000; Abcam, ab180589, Anti-Prostaglandin E Synthase/MPGES-1 antibody [EPR13765]),
23. anti-OPA1 (WB 1:1000; Thermo Fisher, MA5-16149, OPA1 Monoclonal Antibody (1E8-1D9)),
24. anti-PEX5 (WB 1:5000; Novus Biologicals, NBP1-87185),
25. anti-RMDN3 (WB 1:200; Thermo Fisher, PA5-117028, RMDN3 Polyclonal Antibody),

Secondary antibodies:
1. Goat anti-Rabbit IgG (H+L) Secondary Antibody, HRP(WB 1:8000; ThermoFisher, 31460),
2. Rabbit anti-Goat IgG (H+L) Secondary Antibody, HRP(WB 1:10000; ThermoFisher, 31402),
3. Rabbit anti-Mouse IgG (H+L) Secondary Antibody, HRP(WB 1:10000; ThermoFisher, 61-6520),
4. Donkey anti-Rabbit IgG (H+L) Highly Cross-Adsorbed Secondary Antibody, Biotin (IF 1:500; Thermo Fisher, A16039),
5. Goat anti-Rabbit IgG (H+L) Cross-Adsorbed Secondary Antibody, Cyanine3 (IF 1:1000; Jackson Immuno Research Laboratories, 111-165-144),
6. Donkey anti-Rabbit IgG (H+L) Highly Cross-Adsorbed Secondary Antibody, Alexa Fluor™ 568 (IF 1:500; Thermo Fisher, A10042),
7. Donkey anti-Rabbit IgG (H+L) Highly Cross-Adsorbed Secondary Antibody, Alexa Fluor™ 647 (IF 1:500, FACS 1:500; Thermo Fisher, A31573),
8. DyLight™ 405 AffiniPure™ Donkey Anti-Mouse IgG (H+L) (IF 1:300; Jackson Immuno Research Laboratories, 715-475-151),
9. Donkey anti-Mouse IgG (H+L) Highly Cross-Adsorbed Secondary Antibody, Alexa Fluor™ 568 (IF 1:500, FACS 1:500; Thermo Fisher, A10037),
10. DyLight™ 405 AffiniPure™ Donkey Anti-Goat IgG (H+L) (IF 1:300; Jackson Immuno Research Laboratories, 705-475-147)
11. Donkey anti-Goat IgG (H+L) Cross-Adsorbed Secondary Antibody, Alexa Fluor™ 568 (IF 1:500; Thermo Fisher, A11057)
12. Donkey anti-Goat IgG (H+L) Highly Cross-Adsorbed Secondary Antibody, Alexa Fluor™ Plus 647 (IF 1:500, FACS 1:500; Thermo Fisher, A32849),
13. Donkey anti-Rat IgG (H+L) Cross-Adsorbed Secondary Antibody, DyLight™ 550 (IF 1:600; Thermo Fisher, SA5-10027)
14. Alexa Fluor® 647 AffiniPure™ Donkey Anti-Chicken IgY (IgG) (H+L) (IF 1:500, Jackson Immuno Research Laboratories, 703-605-155).
15. Donkey anti-Goat IgG (H+L) Cross-Adsorbed Secondary Antibody, Alexa Fluor™ 405 Plus (IF 1:500; Thermo Fisher, A48258)

Validation

Validation for primary antibodies was provided by the supplier or selected publications as indicated:

1. anti-ABCD1 (Abcam, ab197013): Validated for WB for human and mouse, validated for IF for human and expected to bind murine anti-CD107a (IF 1:200; BioLegend, 121601) samples by the supplier. Relevant citations can be found on supplier website (https://www.abcam.com/en-de/products/primary-antibodies/abcd1-ald-antibody-epr15929-ab197013).

2. anti-ACSL1 (Proteintech, 13989-1-AP): Validated for WB for human and murine samples, for IF for human samples. Relevant citations can be found on supplier website (https://www.ptglab.com/products/ACSL1-Antibody-13989-1-AP.htm).

3. anti-ATGL (CST, 2439): Validated for WB and IF for murine cells by the supplier. Relevant citations can be found on supplier website (https://www.cellsignal.com/products/primary-antibodies/atgl-30a4-rabbit-mab/2439).

4. anti-beta-Actin-HRP (SantaCruz, sc-47778 HRP): Validated for WB for human and murine samples. Relevant citations can be found on supplier website (https://www.scbt.com/p/beta-actin-antibody-c4?productCanUrl=beta-actin-antibody-c4&_requestid=1711860)

5. anti-Calnexin (Proteintech, 10427-2-AP): Validated for IF on human samples by supplier. Relevant citations can be found on supplier website (https://www.ptglab.com/products/CANX-Antibody-10427-2-AP.htm). Usedfor IF on murine samples inthe following publication: Li TY, et al. Tip60-mediated lipin 1 acetylation and ER translocation determine triacylglycerol synthesis rate. Nat Commun. 2018 May 15;9(1):1916. doi: 10.1038/s41467-018-04363-w. PMID: 29765047; PMCID: PMC5953937.

6. anti-Catalase (R&D Systems, AF3398) Validated for WB (human, mouse, rat) and IF (human) by the supplier. Relevant information is provided on the supplier website (https://www.rndsystems.com/products/human-mouse-rat-catalase-antibody_af3398). Specificity for IF in murine cells confirmed by our laboratory through counter staining with peroxisomal markers (data not shown).

7. anti-CD16/CD23 (Thermo Fisher, 14-0161-82): Validated for FACS for murine cells by supplier. Relevant information is provided on the supplier website (https://www.thermofisher.com/antibody/product/CD16-CD32-Antibody-clone-93-Monoclonal/14-0161-82).

8. anti-CD107a (BioLegend, 121601): Validated for FACS and WB with murine samples by supplier. IHC and IF applications validated in publications listed on supplier website (https://www.biolegend.com/en-us/products/purified-anti-mouse-cd107a-lamp-1-antibody-3585?GroupID=BLG4966) such as: Zhou D, et al. Brucella induces unfolded protein response and inflammatory response via GntR in alveolar macrophages. Oncotarget. 2017 Dec 26;9(4):5184-5196. doi: 10.18632/oncotarget.23706. PMID: 29435171; PMCID: PMC5797042.

9. anti-CD107a-A488 (BioLegend, 121607): Validated for FACS on murine samples by supplier. Relevant citations can be found on supplier website (https://www.biolegend.com/en-us/products/alexa-fluor-488-anti-mouse-cd107a-lamp-1-antibody-3588).

10. anti-COX2 (CST, 12282): Validated for IF and WB for murine samples by supplier. Relevant information is provided on the supplier website (https://www.cellsignal.com/products/primary-antibodies/cox2-d5h5-xp-rabbit-mab/12282).

11. anti-cPLA2 (CST, 5249): Validated for WB for human and murine samples by supplier. Relevant citations are provided on the supplier website (https://www.cellsignal.com/products/primary-antibodies/cpla2-d49a7-rabbit-mab/5249).

12. anti-Drp1 (CST, 8570): Validated for WB (human and murine) and IF (human) by supplier. Validation of IF for murine cells in selected publications listed on the supplier website (https://www.cellsignal.com/products/primary-antibodies/drp1-d6c7-rabbit-mab/8570).

13. anti-phospho-Drp1 (S616) (CST, 3455): Validated for WB for human by supplier. Validated for murine samples in selected publications. Relevant citations are provided on the supplier website (https://www.cellsignal.com/products/primary-antibodies/phospho-drp1-ser616-antibody/3455).

14. anti-phospho-Drp1 (S637) (CST, 4867): Validated for WB for rat by supplier. Validated for murine samples in selected publications. Relevant citations are provided on the supplier website (https://www.cellsignal.com/products/primary-antibodies/phospho-drp1-ser637-antibody/4867)

15. anti-FAM73B (Abcam, ab122713): Validated for WB in murine samples in the following publication: Yan MQ, et al. Mitoguardin 1 and 2 promote granulosa cell proliferation by activating AKT and regulating the Hippo-YAP1 signaling pathway. Cell Death Dis. 2023 Nov 27;14(11):779. doi: 10.1038/s41419-023-06312-y. PMID: 38012141; PMCID: PMC10682431.

16. anti-GM130 (BD Bioscience, 610823): Validated for WB and IF for human, mouse and rat by supplier. Relevant information is provided on the supplier website (https://www.bdbiosciences.com/en-us/products/reagents/microscopy-imaging-reagents/immunofluorescence-reagents/purified-mouse-anti-gm130.610823).

17. anti-GNPAT (Proteintech, 14931-1-AP): Validated for WB for human and mouse by supplier. Relevant information is provided on the supplier website (https://www.ptglab.com/products/GNPAT-Antibody-14931-1-AP.htm).

18. anti-HSD17B4 (Novus Biologicals, NBP1-85296): Validated for WB for human and mouse by supplier. Relevant information is provided on the supplier website (https://www.novusbio.com/products/hsd17b4-antibody_nbp1-85296).

19. anti-Hsp60 (CST, 12165): Validated for FACS and IF for human by supplier. Validated for IF for murine samples in publications listed on supplier website (https://www.cellsignal.com/products/primary-antibodies/hsp60-d6f1-xp-rabbit-mab/12165).

20. anti-HSP60 (antibodies.com, A85438): Validated for IF in human cells by supplier. Relevant information is provided on the supplier website (https://www.antibodies.com/hsp60-antibody-a85438).

21. anti-MFN2 (Abcam, ab124773): Validated for WB for human, mouse and rat by supplier. Relevant information is provided on the supplier website (https://www.abcam.com/products/primary-antibodies/mitofusin-2-antibody-niar164-ab124773.html).

22. anti-MPGES1 (Abcam, ab180589): validated for WB for human and mouse by supplier. Relevant information is provided on the supplier website (https://www.abcam.com/products/primary-antibodies/prostaglandin-e-synthasempges-1-antibody-epr13765-ab180589.html).

23. anti-OPA1 (Thermo Fisher, MA5-16149): Validated for WB in human, mouse, rat, hamster by supplier. Relevant information is provided on the supplier website (https://www.thermofisher.com/antibody/product/OPA1-Antibody-clone-1E8-1D9-Monoclonal/MA5-16149).

24. anti-PEX5 (Novus Biologicals, NBP1-87185): Validated for WB in human and mouse by supplier. Relevant information is provided on the supplier website (https://www.novusbio.com/products/pex5-antibody_nbp1-87185).

25. anti-RMDN3 (Thermo Fisher, PA5-117028): Validated for WB in human and mouse by supplier. Relevant information is provided on the supplier website (https://www.thermofisher.com/antibody/product/RMDN3-Antibody-Polyclonal/PA5-117028)

Eukaryotic cell lines

Policy information about cell lines and Sex and Gender in Research

Cell line source(s)	Platinum-E and HEK 293T were sourced from R. Grosschedl (MPI-IE, Freiburg).
Authentication	Platinum-E and HEK 293T cells were not authenticated.
Mycoplasma contamination	Cell lines are regularly checked and tested negative for Mycoplasma infection.
Commonly misidentified lines (See ICLAC register)	No commonly misidentified cell lines were used in this study.

Animals and other research organisms

Policy information about studies involving animals; ARRIVE guidelines recommended for reporting animal research, and Sex and Gender in Research

Laboratory animals	C57BL/6 mice (RRID: IMSR_JAX:000664), Drp1 -/- mice (strain C57BL6/J Cx3cr1tm1.1(cre)Jung Dnm1ltm1.1Miha), Pex5 -/- mice (strain C57BL6/J Lyz2tm1(cre)Ifo Pex5tm1(Pec)Baes), Opa1 -/- mice (C57BL6/J Lyz2tm1(cre)Ifo Opa1tm1.1Hise). C57BL/6J (JAX, #000664), Pex5fl/fl mice (B6J.129-Pex5tm1Pec/BaesJ; JAX, #031665) were purchased from the Jackson Laboratories. Opa1fl/fl and Drp1fl/fl mice were a kind gift from Erika Pearce. Mice were housed under controlled conditions, namely 20–24°C, 45–65% relative humidity, and 14:10 h light-dark cycle. Food was available ad libitum for all animals ("Mouse Breeding, High Energy", ssniff, V1185-300). Animal breeding and husbandry were performed in accordance with the guidelines provided by the Federation of European Laboratory Animal Science Association and by German authorities and the Regierungspräsdium Az. 35-9185.64/1.1. For tissue isolation, animals were euthanized by carbon dioxide asphyxiation prior to tissue removal in compliance to § 4, paragraph 3 of the German Animal Protection Act. Age- (8-25 weeks) and sex-matched male and female animals were used for bone marrow isolations of knock-out strains. Male (8-12 weeks) C57BL/6J mice were used for bone marrow isolations for all other experiments. Aged (114-116 weeks) and young (8-15 weeks) female C57BL/6J mice were used to isolate peritoneal macrophages. Aged mice were obtained through the shared animal program for animal welfare at the Max Planck Institute of Immunobiology and Epigenetics.
Wild animals	No wild animals were used for this study.
Reporting on sex	Sex was not considered for the study design. Male and female animals were assigned to the experiments at random. No sex-based analysis were performed.
Field-collected samples	No field-collected samples were used for this study.
Ethics oversight	Animal breeding and husbandry were performed in accordance with the guidelines provided by the Federation of European Laboratory Animal Science Association and by German authorities and the Regierungspräsdium Az. 35-9185.64/1.1. For tissue isolation, animals were euthanized by carbon dioxide asphyxiation prior to tissue removal in compliance to § 4, paragraph 3 of the German Animal Protection Act.

Note that full information on the approval of the study protocol must also be provided in the manuscript.

Flow Cytometry

Plots

Confirm that:

☒ The axis labels state the marker and fluorochrome used (e.g. CD4-FITC).

☒ The axis scales are clearly visible. Include numbers along axes only for bottom left plot of group (a 'group' is an analysis of identical markers).

☒ All plots are contour plots with outliers or pseudocolor plots.

☒ A numerical value for number of cells or percentage (with statistics) is provided.

Methodology

Sample preparation	For flow cytometry analysis, primary bone marrow-derived macrophages (BMDMs) were used. To measure mitochondrial parameters, BMDMs were stained with 100 nM MitotrackerGreen alone or in combination with 50 nM TMRM. Cells were subsequently washed three times with warm BMDM medium, harvested with 20 mM EDTA and then incubated for 5 min with DAPI (0.5 µg/ml) in PBS. To measure mitochondrial ROS, cells were treated with 5 µM MitoSOX in phenol-fee BMDM medium (10 % FCS, 1% P/S). For cellular ROS measurement, BMDMs were treated with 0.5 µM CellROX in phenol-free BMDM

medium for 45 min at 37 °C. For both treatments, cells were subsequently washed three times with prewarmed phenol-fee BMDM medium and treated as mentioned above.
To validate the expression of maker proteins used for spectral imaging, activated and control BMDMs were collected with 20 mM EDTA, incubated for 10min on ice with Live Dead Fixable Viability eFluor780 or eFluor450 (1:1000) in PBS and fixed using the Foxp3 transcription factor staining buffer set (eBioscience, 00552300). Samples were then incubated in wash buffer containing 5% FCS and CD16/32 blocking antibodies (1:1000, BD Biosciences) for 1h at RT and subsequently stained with primary antibodies in wash buffer for 1 h at RT. Secondary antibody staining was performed in wash buffer for 1h at RT.
All samples were measured on the BD LSR Fortessa cell analyser (BD Biosciences) data were analysed in FlowJo (BD Biosciences, v.10.7.1).

Instrument

BD LSR Fortessa cell analyser (BD Biosciences)

Software

BD FACS Diva (BD Biosciences v8.0.1) for data collection, FlowJo (BD Biosciences, v.10.7.1)

Cell population abundance

N/A

Gating strategy

The BMDM population was gated on single cells according to FSC-H/FSC-W. Living cells were defined as DAPI- or Fixable Viability Dye-negative populations. The MFI (geometric mean) of used dyes or antibody stainings was measured for the living population and averaged across all technical replicates. Depicted in the figures is the average MFI of independent experiments as indicated in the figure legends.

☒ Tick this box to confirm that a figure exemplifying the gating strategy is provided in the Supplementary Information.
