# Supplementary figures and images for: Functional multi-organelle units control inflammatory lipid metabolism of macrophages
Source: Nat Cell Biol. 2024 Jul 5;26(8):1261–73. doi: 10.1038/s41556-024-01457-0 (PMC11321999; doi:10.1038/s41556-024-01457-0)

**Supplementary Figure 1**  
Source Data corresponding to Extended Data Fig. 2, Western Blot images

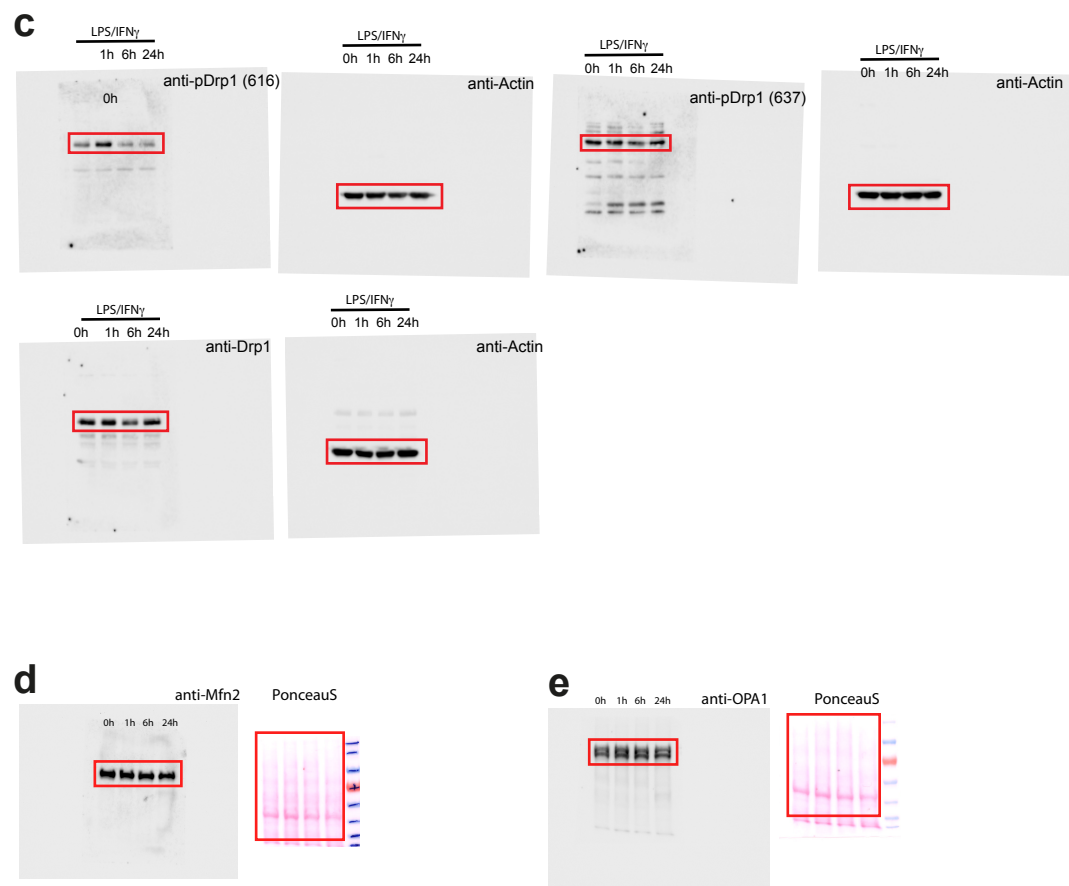

Supplement: Supplementary file 13 — Unprocessed WBs. [file 41556_2024_1457_MOESM13_ESM.pdf]

**Supplementary Figure 2**  
Source data corresponding to ED Fig. 7, Western Blot images

**a**

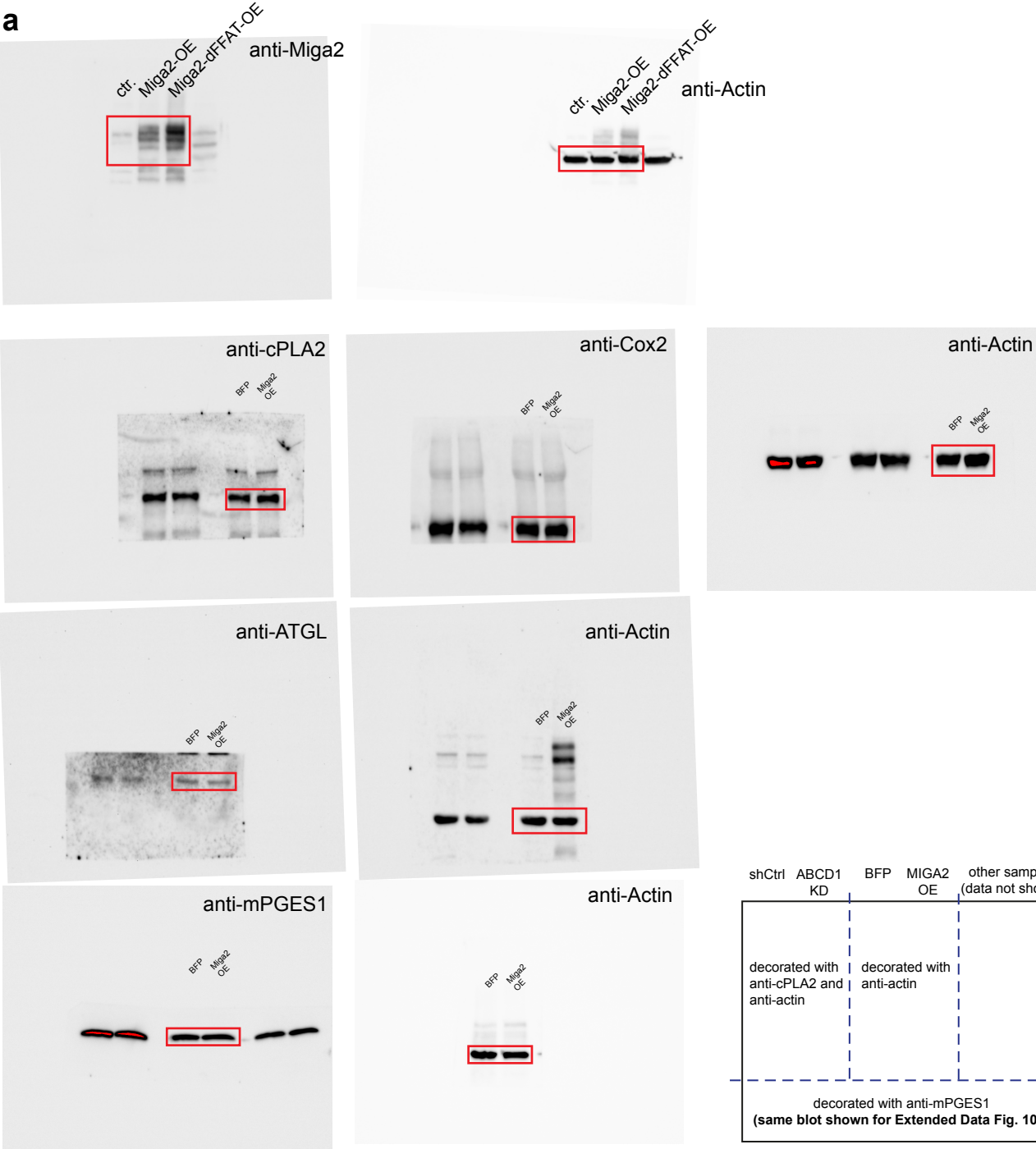

Supplement: Supplementary file 19 — Unprocessed WBs. [file 41556_2024_1457_MOESM19_ESM.pdf]

**Supplementary Figure 3**  
Source data corresponding to ED Fig. 8, Western Blot images

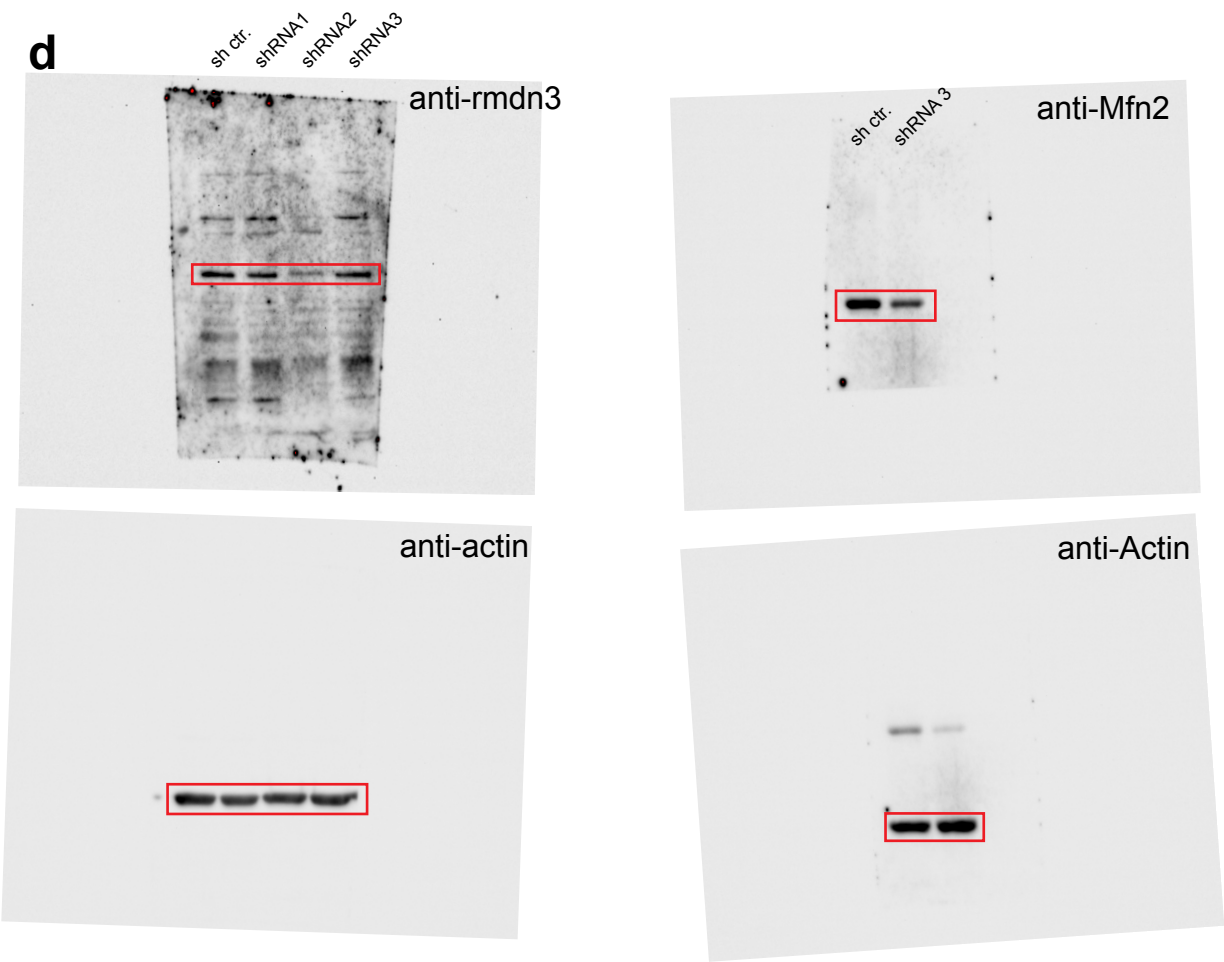

Supplement: Supplementary file 21 — Unprocessed WBs. [file 41556_2024_1457_MOESM21_ESM.pdf]

# Supplementary Figure 4

Source Data corresponding to ED Data Figure 9, Western Blot images

**a**

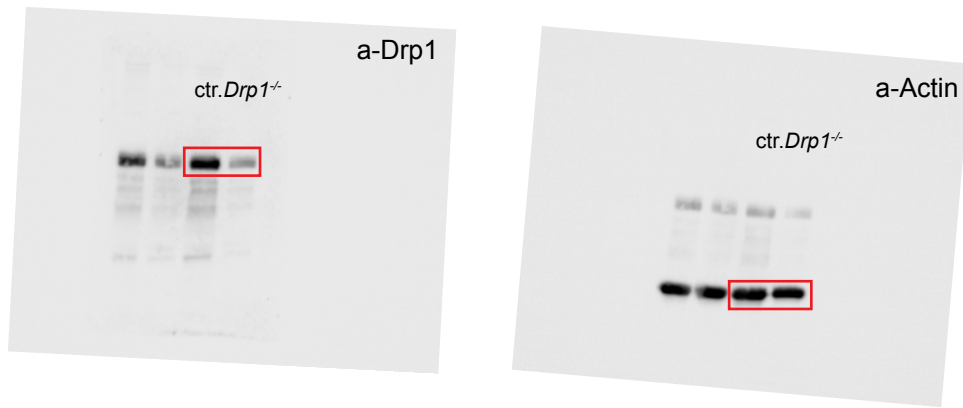

**c**

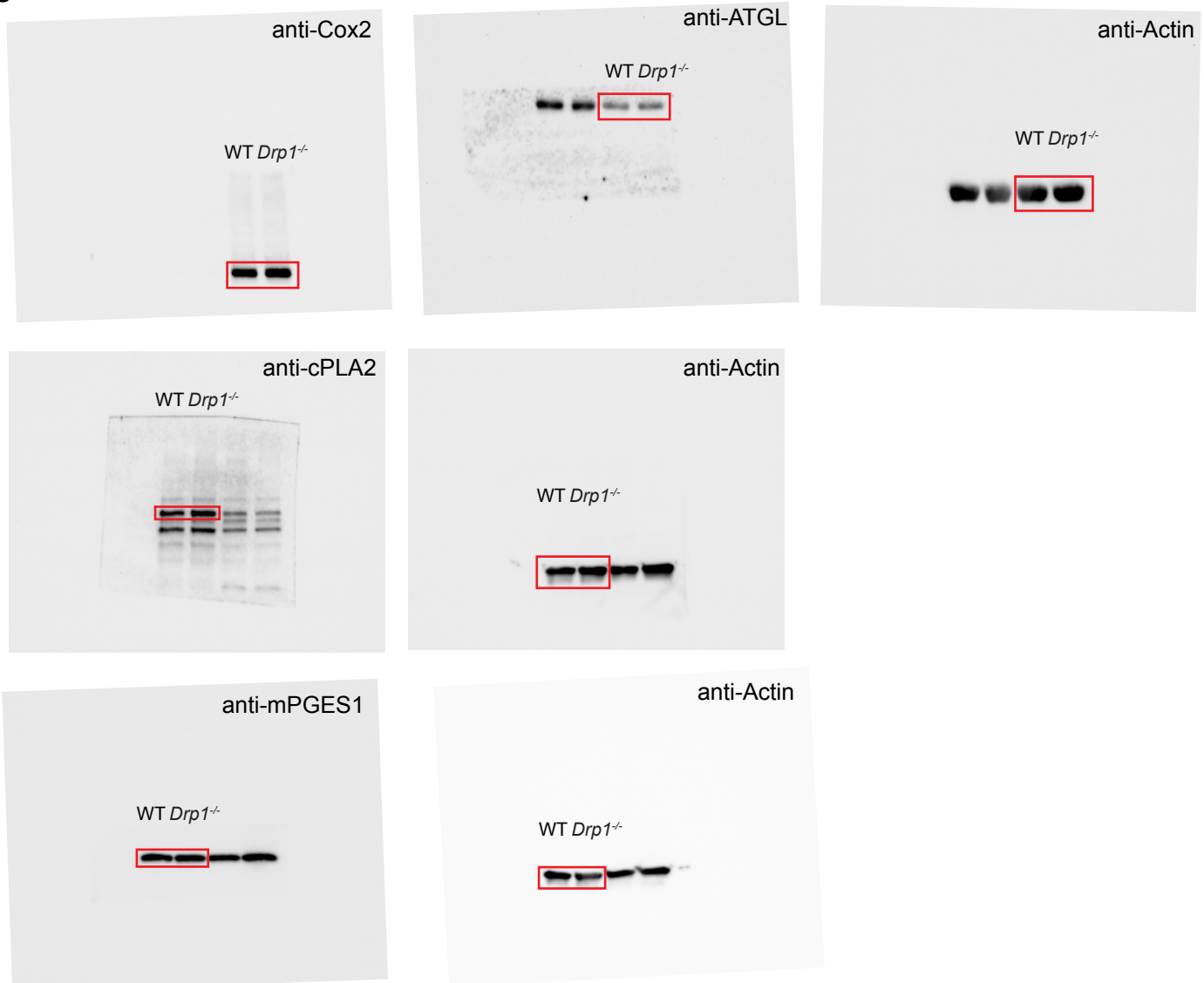

**k**

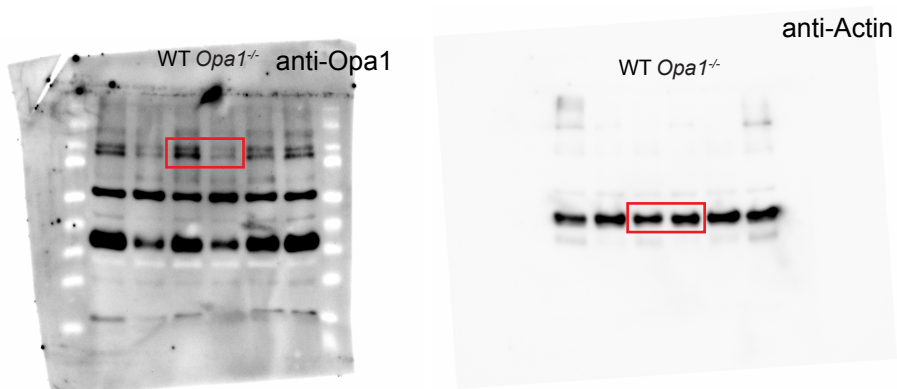

Supplement: Supplementary file 23 — Unprocessed WBs. [file 41556_2024_1457_MOESM23_ESM.pdf]

# Supplementary Figure 5

Source Data for ED Figure 9, Western Blot images

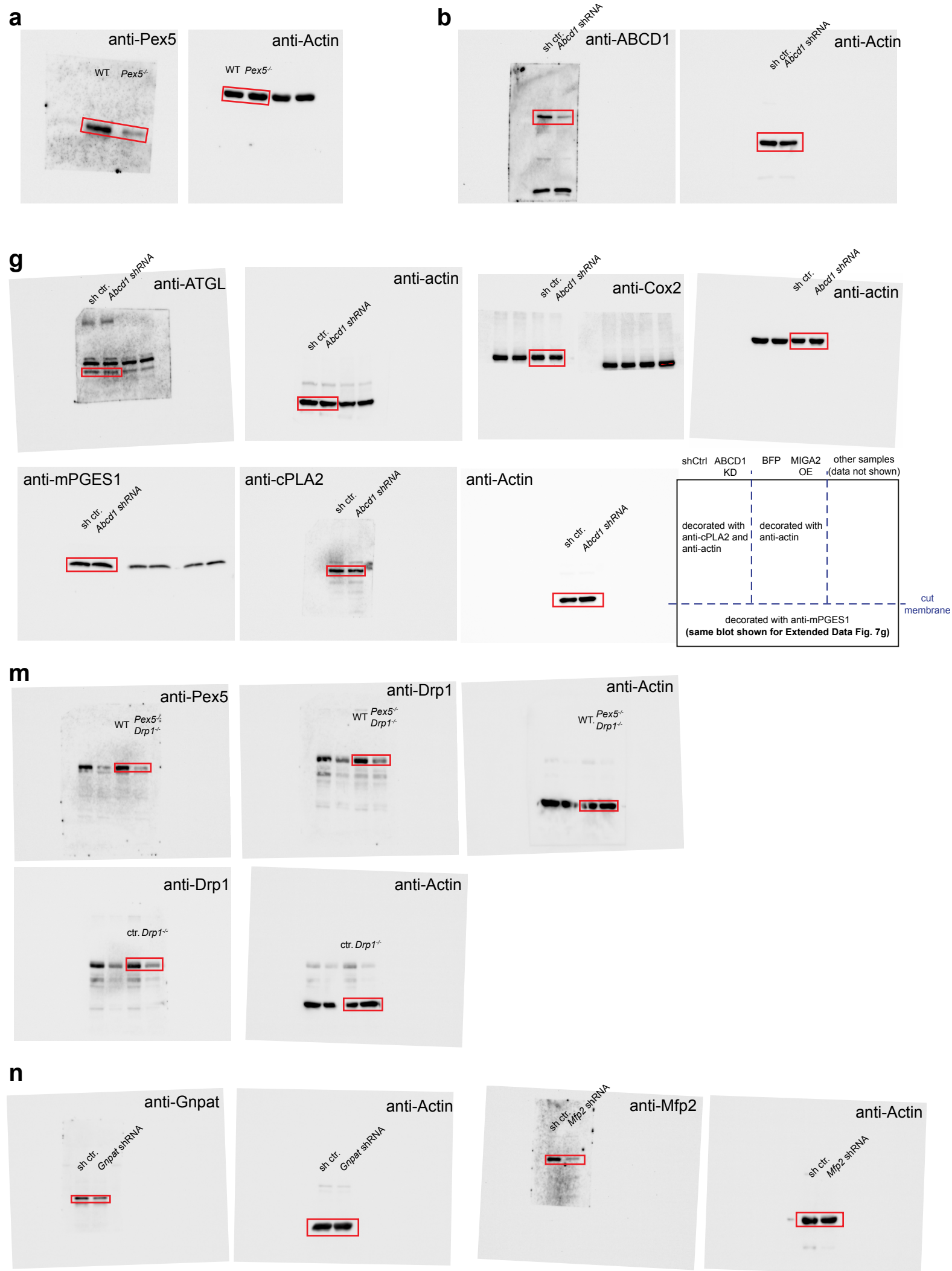

Supplement: Supplementary file 25 — Unprocessed WBs. [file 41556_2024_1457_MOESM25_ESM.pdf]
